# Impact of Biogenic and Chemogenic Selenium Nanoparticles on Model Eukaryotic Lipid Membranes

**DOI:** 10.1021/acs.langmuir.3c00718

**Published:** 2023-07-18

**Authors:** Elena Piacenza, Kevin Sule, Alessandro Presentato, Frieda Wells, Raymond J. Turner, Elmar J. Prenner

**Affiliations:** † Department of Biological, Chemical and Pharmaceutical Science and Technologies, University of Palermo, Viale delle Scienze, Ed. 16, 90128 Palermo, Italy; ‡ Department of Biological Sciences, University of Calgary, 2500 University Dr. NW, Alberta, Calgary T2N 1N4, Canada

## Abstract

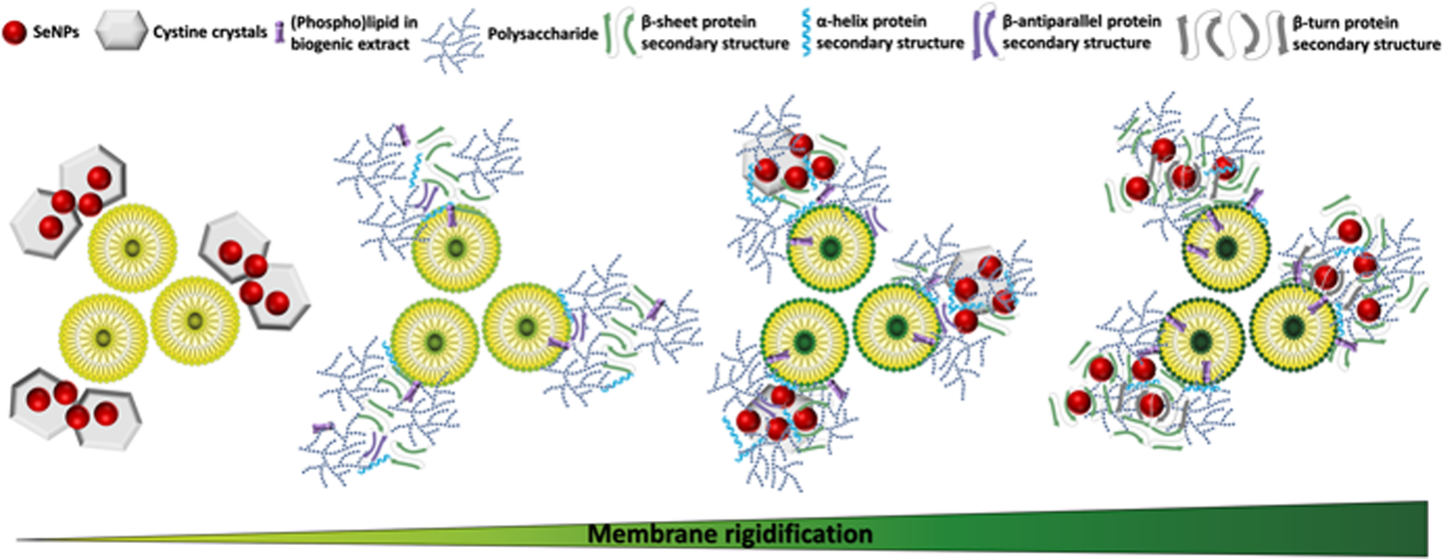

Microbial nanotechnology is an expanding research area devoted to producing biogenic metal and metalloid nanomaterials (NMs) using microorganisms. Often, biogenic NMs are explored as antimicrobial, anticancer, or antioxidant agents. Yet, most studies focus on their applications rather than the underlying mechanism of action or toxicity. Here, we evaluate the toxicity of our well-characterized biogenic selenium nanoparticles (bSeNPs) produced by the *Stenotrophomonas maltophilia* strain SeITE02 against the model yeast *Saccharomyces cerevisiae* comparing it with chemogenic SeNPs (cSeNPs). Knowing from previous studies that the biogenic extract contained bSeNPs in an organic material (OM) and supported here by Fourier transform infrared spectroscopy, we removed and incubated it with cSeNPs (cSeNPs_OM) to assess its influence on the toxicity of these formulations. Specifically, we focused on the first stages of the eukaryotic cell exposure to these samples—i.e., their interaction with the cell lipid membrane, which was mimicked by preparing vesicles from yeast polar lipid extract or phosphatidylcholine lipids. Fluidity changes derived from biogenic and chemogenic samples revealed that the bSeNP extract mediated the overall rigidification of lipid vesicles, while cSeNPs showed negligible effects. The OM and cSeNPs_OM induced similar modifications to the bSeNP extract, reiterating the need to consider the OM influence on the physical–chemical and biological properties of bSeNP extracts.

## Introduction

Over the past 40 years, nanotechnology greatly impacted both fundamental and applied research, as materials scaled down to the nano range (i.e., nanomaterials—NMs) feature emphasized physical–chemical properties rather than bulk ones.^1^ For instance, metal and metalloid [henceforth metal(loid)] NMs exhibit unique electronic, optical, catalytic, and biological properties that make them valuable for a variety of applications.^2,3^ Many synthetic procedures have been developed to produce metal(loid) NMs. Yet, most of these approaches often involve hazardous chemicals, dangerous operational conditions, and toxic waste that needs to be disposed of.^4^ Besides, chemical and physical methods must account for and overcome the tendency of NMs to aggregate by using toxic and expensive chemicals as capping agents to ensure NM thermodynamic stability.^5^ Microbial nanotechnology (i.e., the exploitation of microorganisms to transform toxic chemicals into metal(loid) NMs) represents an appealing alternative to most physical or chemogenic procedures, as it allows obtaining thermodynamically stable NMs in an eco-friendly fashion.^6^

Among metal(loid) NMs, those based on selenium (Se) have gained momentum. Indeed, this element is biocompatible and a known essential micronutrient that, due to its physical–chemical versatility, finds application in diverse fields ranging from biomedicine to renewable energy production.^4,7^ The microbial production of SeNMs provides the biotechnological benefit of recovering a rare earth element such as Se in a nontoxic form, gaining, at the same time, suitable products for developing novel technologies.^8−10^ Specifically, microorganisms can synthesize stable biogenic SeNMs (bSeNMs) of different sizes and morphologies with good antimicrobial, anticancer, antioxidant, and photoluminescence properties.^8−11^

Despite the known biological activity of bSeNMs, understanding their interaction and effect on eukaryotic systems is still in its infancy. Thus, the present study explored the susceptibility of the yeast model *Saccharomyces cerevisiae* against the biogenic Se nanoparticle (bSeNP) extract produced by the environmental isolate *Stenotrophomonas maltophilia* strain SeITE02and chemogenic SeNPs (cSeNPs), both of which were characterized through transmission electron microscopy (TEM), ζ-potential measurements, and Fourier transform infrared spectroscopy in attenuated total reflectance (ATR-FTIR) mode. Considering that the first interaction of bioactive compounds and materials is with the cell membrane, we focused our study on the effects of SeNPs on model lipid membranes. To this aim, we first explore vesicles made of the complex yeast polar lipid extract before examining vesicles of defined lipids (i.e., 1-palmitoyl-2-oleoyl-sn-glycero-3-phosphocholine (POPC) and 1,2-dimyristoyl-*sn*-glycero-3-phosphocholine (DMPC)). Our approach here used fluorescence generalized polarization (GP) and anisotropy ⟨*r*⟩, which gave insights into membrane fluidity changes at the interphase and the hydrophobic core.^12,13^

## Experimental Section

### Biogenic and Chemogenic SeNP Synthesis and Organic Material Recovery

bSeNP extract and cSeNPs were produced as described by Piacenza and co-workers.^11^ Briefly, bSeNPs were synthesized by *Stenotrophomonas maltophilia* SeITE02 cells during 48 h growth in Luria Bertani broth in the presence of 500 μM Na_2_SeO_3_. Since previous studies showed that SeITE02 cells secreted SeNPs,^14,15^ the obtained cell-free spent medium was recovered through a centrifugation step (8000*g* for 15 min) and filtered using 0.2 μm Filtropur (Sarstedt). The latter was centrifuged at 12,000*g* for 10 min to pellet bSeNPs, which were then resuspended in 500 μL of sterile distilled water generating the bSeNP extract.

The organic material (OM) associated with the bSeNP was obtained from the bSeNP extract through centrifugation (12,000*g* for 10 min) and the recovery of the SeNP-free supernatant.^11^

The cSeNPs were synthesized as reported by Li et al.^16^ using a 1:3 molar ratio of Na_2_SeO_3_ (100 mM stock solution) and l-cysteine (50 mM stock solution) to obtain NPs comparable in size with those biogenic.^11^ Moreover, the preparation of cSeNPs was centrifuged (12,000*g* for 10 min), resuspended in the OM solution, and incubated for 16 h at room temperature (henceforth indicated as cSeNPs_OM).^11^

Biogenic and chemogenic SeNPs were extensively produced following these procedures and previously characterized for size (average diameter of ca. 50 nm),^11^ shape—spherical,^11,14,16−21^ polydispersity,^11,14,16−23^ thermodynamic stability,^14,16,18−23^ and optical and photoluminescence properties.^11,15^ Similarly, earlier studies reported on the existence of an OM embedding bSeNPs synthesized from the SeITE02 strain.^11,14,15,17−19,21,23^ The physical–chemical characterization of this distinguished trait highlighted the presence of proteins, carbohydrates, amphiphilic substances, and lipids, with the latter the most represented.^11,14,15,19,23^ Finally, SeNPs within cSeNPs_OM featured a comparable size (ca. 50 nm), shape, thermodynamic stability, and optical and photoluminescence properties to those obtained only using l-cysteine as a stabilizing agent.^11^

The concentration of bSeNP extract, cSeNPs, OM, and cSeNPs_OM was estimated as wet weight (expressed as μg/mL) and, when necessary, samples were opportunely diluted with distilled water to obtain 10, 50, or 100 μg/mL as final concentrations. All of the samples were stored at 4 °C before their use.

### Transmission Electron Microscopy (TEM)

TEM analysis of chemogenic and biogenic SeNPs was performed as indicated by Piacenza and colleagues^22^ by mounting 5 μL of each sample onto carbon-coated copper grids (CF-300 CU Electron Microscopy Sciences). The samples were air-dried and analyzed using a Hitachi H7650 TEM. The average diameter of SeNPs was calculated by measuring 100 randomly chosen NPs using ImageJ software.

### ζ-Potential Measurements

ζ-Potential measurements were performed on 1 mL aliquots of the bSeNP extract, cSeNPs, OM, and cSeNPs_OM to gain information regarding their surface charge, as described by Piacenza and colleagues.^22,24^ Measurements were carried out in triplicate (*n* = 3, 100 scans each, acquisition time 30 s) under isothermal conditions (*T* = 25 °C) using a Zen 3600 Zetasizer Nano ZS (Malvern Instruments). ζ-Potential values are reported as average values with standard deviation.

### Fourier Transform Infrared Spectroscopy in Attenuated Total Reflectance (ATR-FTIR) Mode

ATR-FTIR spectroscopy was performed in triplicate (*n* = 3) on the bSeNP extract, cSeNPs, OM, and cSeNPs_OM as described by Piacenza and colleagues^24^ through a Bruker Vertex70 Advanced Research FTIR spectrometer equipped with a platinum ATR and a diamond crystal. ATR-FTIR spectra (4000–70 cm^–1^ range, 2 cm^–1^ lateral resolution, 200 scans) were analyzed by using OPUS7.5 (Bruker Instruments) and OriginPro 2016 software. Spectral deconvolutions by nonlinear least-square fitting were performed in the 1780–1420 and 1420–940 cm^–1^ regions of the bSeNP extract, OM, and cSeNPs_OM acquired spectra using OriginPro 2016 software. Peak integrals of interest for further analysis were duly normalized to better highlight differences between samples. Peak integrals referring to CH*_x_* stretching vibrations of lipids and amide A and B of proteins were normalized against the integral calculated in the 3600–2500 cm^–1^ region of the spectra. Peak areas attributed to amide I, II, and III of proteins, −CO stretching (ca. 1740 cm^–1^), and bending vibrations of lipids (1460–1360 cm^–1^) and polysaccharides were normalized against the integral of the 1800–900 cm^–1^ region.

### *Saccharomyces cerevisiae* Susceptibility against SeNPs

The susceptibility of *S. cerevisiae* against bSeNP extract and cSeNPs was evaluated using the Calgary biofilm device (CBD—sold as the MBEC physiology and Genetics assay by Innovotech Inc.) following the procedure previously used by Harrison and colleagues.^25^ The CBD peg lid was immersed in a sterile solution of 1% l-lysine to improve yeast cell adhesion and incubated for 16 h at room temperature. *S. cerevisiae* cells were cultured in tryptic soy broth (TSB, EMD chemicals inc.) overnight (ca. 16 h) and used for a starting culture matching 1 McFarland standard (ca. 3.0 × 10^8^ CFU/mL). Aliquots (100 μL) of this culture were placed in each well of a microtiter plate containing 10, 50, or 100 μg/mL of bSeNP extracts, cSeNPs, OM, or cSeNPs**_**OM and TSB to reach 180 μL total volume. The peg lid was placed on the microtiter plate, and the device was incubated for 24 h at 37 °C with shaking (125 rpm). After incubation, colony forming units (CFUs) for *S. cerevisiae* planktonic cells were estimated using the microtiter plate bottom through serial dilution and plating onto TSB agar. Yeast biofilms formed onto the CBD peg lid were rinsed twice in phosphate buffered saline [PBS; composed (g/L) of sodium chloride (NaCl; 8), potassium chloride (KCl; 0.2), disodium hydrogen phosphate (Na_2_HPO_4_; 1.44), and potassium dihydrogen phosphate (KH_2_PO_4_; 0.24)], pH 7.4, to remove loosely adherent cells and then transferred to a new microtiter plate containing fresh TSB. *S. cerevisiae* biofilms were detached from the CBD pegs through a sonication step (30 min), and biofilm CFUs were evaluated following the same method described for planktonic cells. The susceptibility experiments were performed in triplicate (*n* = 3). The data are reported as kill curves, displaying the average number of surviving CFUs as a function of added SeNPs with standard deviation.

### Liposome Preparation of Defined Lipid Composition

Large unilamellar vesicles (LUVs) with defined lipid composition and size were prepared from POPC (1-palmitoyl-2-oleoyl-*sn*-glycero-3-phosphocholine) and DMPC (1,2-dimyristoyl-*sn*-glycero-3-phosphocholine) as well as *S. cerevisiae* polar lipid extract (Avanti Lipids). Yeast (YE) polar lipid extract composition of known components: l-α-glycerophosphoryl(choline) 0.18% (wt/wt), phosphatidylcholine 29.15%, lyso-phosphatidylcholine 1.35%, phosphatidylethanolamine 7.83%, lyso-phosphatidylethanolamine 4.12%, phosphatidylinositol 26.21%, phosphatidylserine 7.86%, phosphatidic acid 1.97%, phosphatidylglycerol 0.68%, and unknown 0.86%.

Lipid films were prepared by accurately weighing lipid powders using a Sartorius Microbalance MC5 (Göttingen, Germany) in a clean borosilicate vial (VWR, Mississauga, ON) and co-dissolving in 7:3 chloroform/methanol with either laurdan or diphenylhexatriene in a 1:500 dye/lipid molar ratio. This ratio was chosen to provide a sufficient signal of the dye without any perturbations to membrane properties.^26^ The organic solvent was purged using argon gas, followed by an overnight vacuum to ensure the removal of any solvent traces. Lipid films were re-hydrated with 100 mM NaCl (pH 7.4) with subsequent vortexing, sonication, and at least 5 freeze–thaw cycles to generate multilamellar vesicles (MLVs). This procedure also ensured the complete removal of the lipid film from the surface of the glass vial. LUVs were made by passing the MLVs through a 200 nm nucleopore polycarbonate filter using an extruder (Avanti mini extruder, Alabaster, AL). The MLV suspension was passed between 2 gas-tight syringes at least 20 times to ensure uniform size distribution of LUVs. For each lipid system, MLVs were extruded above the phase transition temperature (*T*_m_). After extrusion, the phospholipid concentration was determined using a phosphate assay, according to Ames.^27^ Moreover, the liposome suspensions were re-suspended in pH**-**corrected unbuffered 100 mM NaCl solution to avoid any unspecific interaction of the analyzed samples.

The obtained LUVs were characterized through DLS and ζ-potential measurements using a Zen 3600 Zetasizer Nano ZS (Malvern Instruments). Measurements were performed on 1 mL aliquots of LUVs in triplicate (*n* = 3) as described in the ζ-Potential Measurements section. Hydrodynamic diameter (*d*_H_) and ζ-potential are reported as average values with standard deviation.

### Fluorescence Laurdan Generalized Polarization (GP) and Diphenylhexatriene (DPH) Anisotropy ⟨*r*⟩

Fluorescence measurements were conducted using POPC, DMPC, and *S. cerevisiae* polar lipid extract systems exposed to bSeNP extract (10, 50, and 100 μg/mL), cSeNPs (10, 50, and 100 μg/mL), OM (100 μg/mL), and cSeNPs_OM (100 μg/mL) to address changes in membrane fluidity. Specifically, laurdan (6-dodecanoyl-2-dimethylaminonapthalene, Molecular Probes, Eugene, OR) is a solvent-sensitive dye that can detect changes in membrane properties in response to its surrounding solvent environment.^12,26^ We have used this approach on vesicles composed of polar lipid extracts,^28^ and this method is even suitable for analyzing effects on trace concentrations of target lipids.^29^ The hydrophobic side chain of laurdan ensures its insertion into membranes, allowing evaluating fluidity changes in the interphase and headgroup regions of the lipids in the membrane. Fluorescence measurements for laurdan generalized polarization (GP) were performed using a Cary Eclipse fluorimeter (Agilent Technologies, Santa Clara, CA) at an excitation wavelength of 340 nm, measuring emission at both 440 and 490 nm as an average of 3 measurements and using an excitation and emission bandpass of 5 nm.

For fluorescence anisotropy experiments ⟨*r*⟩, the probe diphenylhexatriene (DPH, Sigma-Aldrich, Oakville, ON) is used. DPH is very hydrophobic and readily inserts deep into membranes, where it reports on the fluidity of the hydrophobic core. The DPH has a sensitive polarization response to fatty acyl tail order and orientation.^13^ We have previously used the combination of GP and ⟨*r*⟩ to compare fluidity changes at the interphase (GP) with effects in the hydrophobic core.^30^ For this experiment, fluorescence measurements were carried out using a Shimadzu fluorimeter (Mandel, Guelph, ON) at an excitation wavelength of 360 nm and emission scans at 430 nm. The fluorimeter was equipped with polarizers to select for polarized light in both the vertical (*I*_VV_) and horizontal (*I*_VH_) directions.

For these fluidity experiments, 0.1 mM LUVs in small-volume quartz cuvettes (Starna Scientific Ltd, Atascadero, CA) were amended with SeNP-containing or -derived samples (10, 50, or 100 μg/mL) and incubated for 5 min before any measurements. The temperature was controlled to ±0.1 °C by using a circulating water bath (Agilent Technologies, Santa Clara, CA). Measurements were conducted in a temperature range of 10–50 °C, depending on the *T*_m_ of each lipid system. Samples were equilibrated for at least 1 min/1 °C change in temperature.

The statistical significance of the results obtained for either GP or ⟨*r*⟩ variations was evaluated by performing an unpaired Student’s *t*-test (OriginPro software package) using the 95% confidence interval. The statistical significance of the GP and ⟨*r*⟩ variations between datasets was considered when *p* < 0.05.

## Results and Discussion

### Physical–Chemical Characterization of bSeNP Extract, cSeNPs, OM, and cSeNPs_OM

Among the elevated number of SeNP-producing microorganisms, the aerobic Gram-negative *S. maltophilia* SeITE02 strain isolated from the rhizosphere of the Se hyperaccumulator plant *Astragalus bisulcatus*(31) is of great interest. This bacterium well tolerates selenium oxyanions such as selenite and produces spherical SeNPs secreted in the extracellular milieu during bacterial growth.^14,15^

The formulations used in the present manuscript were extensively characterized in our earlier reports (see the Biogenic and Chemogenic SeNP Synthesis and Organic Material Recovery section). Nevertheless, TEM imaging, ζ-potential measurements, and ATR-FTIR spectroscopy were performed to verify SeNP size and shape, alongside the OM presence within these samples.

#### Transmission Electron Microscopy

**Figure 1:**
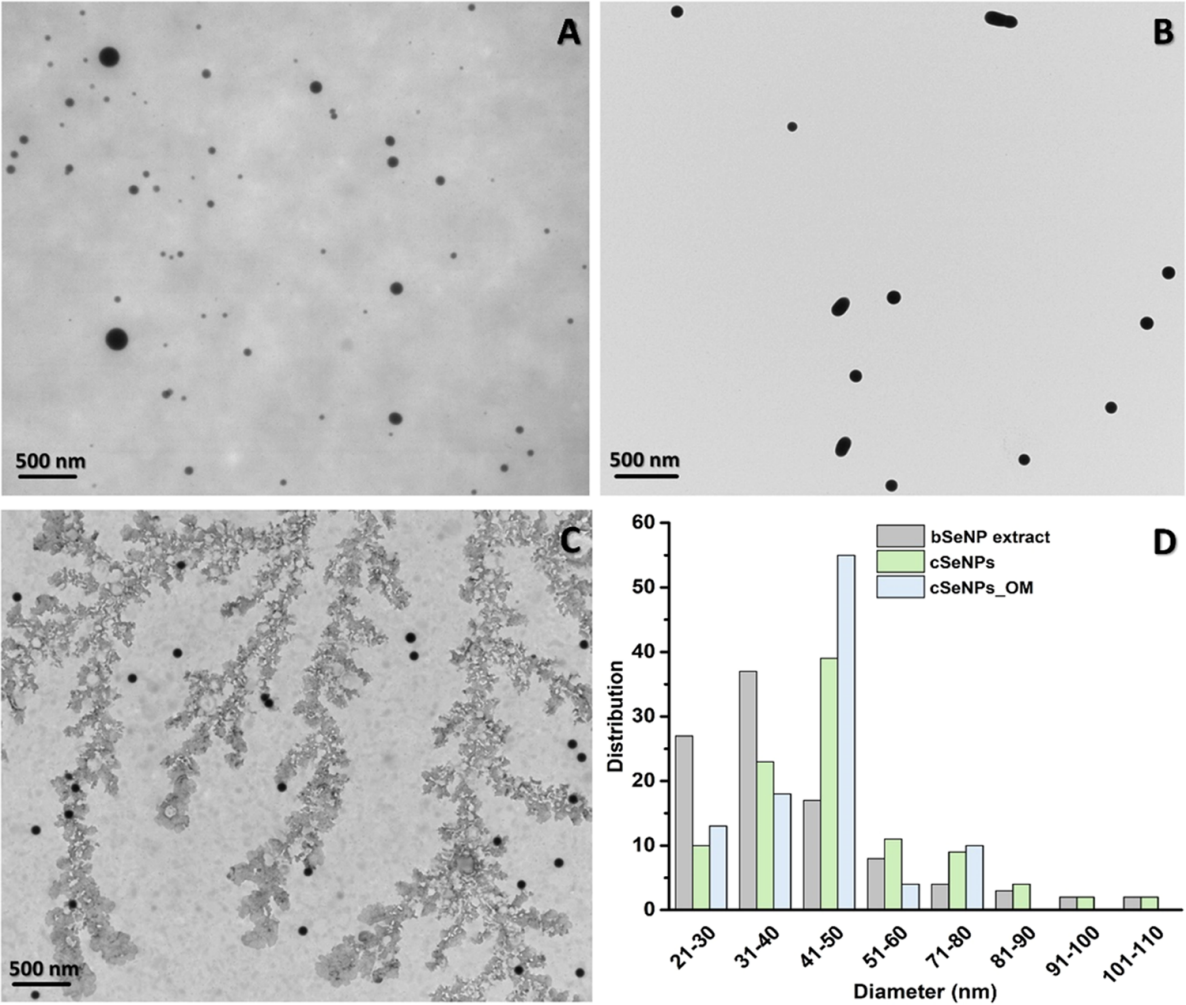
Transmission electron microscopy images of (A) the bSeNP extract, (B) cSeNPs, and (C) cSeNPs_OM. (D) Size distribution of SeNPs.

The bSeNP extract recovered from *S. maltophilia* SeITE02 cells featured regular and spherical SeNPs (Figure 1A) with an average size of 40 ± 15 nm, in line with our earlier report.^11^ cSeNPs were comparable in size (48 ± 19 nm) and morphology with those biogenic, yet some larger and nonspherical NPs were detected (Figure 1B). On the opposite, the bSeNP extract revealed higher thermodynamic stability than cSeNPs, as no change in morphology was detected in the former (Figure 1A). The latter feature of the biogenic extract derives from the presence of a slight electron-dense material, likely of organic nature (i.e., OM) that surrounds SeNPs. The importance of the OM as an NP stabilizer was confirmed by TEM micrographs of cSeNPs incubated with the OM (cSeNPs_OM), which revealed regular and spherical SeNPs with an average size of 45 ± 8 nm and without signs of aggregation (Figure 1C), corroborating the ability of this material to interact with SeNPs.

#### Surface Charge

All samples featured negative ζ-potential values (Table 1), indicating the adsorption of charged functional groups onto the SeNP surface.

**Table 1:** ζ-Potential Values of the bSeNP Extract, cSeNPs, OM, and cSeNPs_OM

sample	ζ-potential (mV)
bSeNP extract	–24 ± 2
cSeNPs	–56 ± 2
OM	–22 ± 1
cSeNPs_OM	–20 ± 3

cSeNPs displayed the lowest surface charge, which derived from the presence of cystine and l-cysteine-related species alongside the excess of selenite in the aqueous solution. In contrast, the three OM-containing samples showed similar ζ-potential values that aligned with those previously reported in our studies.^19,23^ This outcome indicated that biomolecules deriving from the *S. maltophilia* SeITE02 strain effectively embedded cSeNPs contributing, through electrostatic interactions, to their overall stabilization. Besides biomolecules’ presence, the increased surface charge observed for cSeNPs_OM compared with cSeNPs may relate to the removal of selenite excess in the former occurring during its preparation (i.e., centrifugation step).

#### ATR-FTIR Spectroscopy

**Figure 2:**
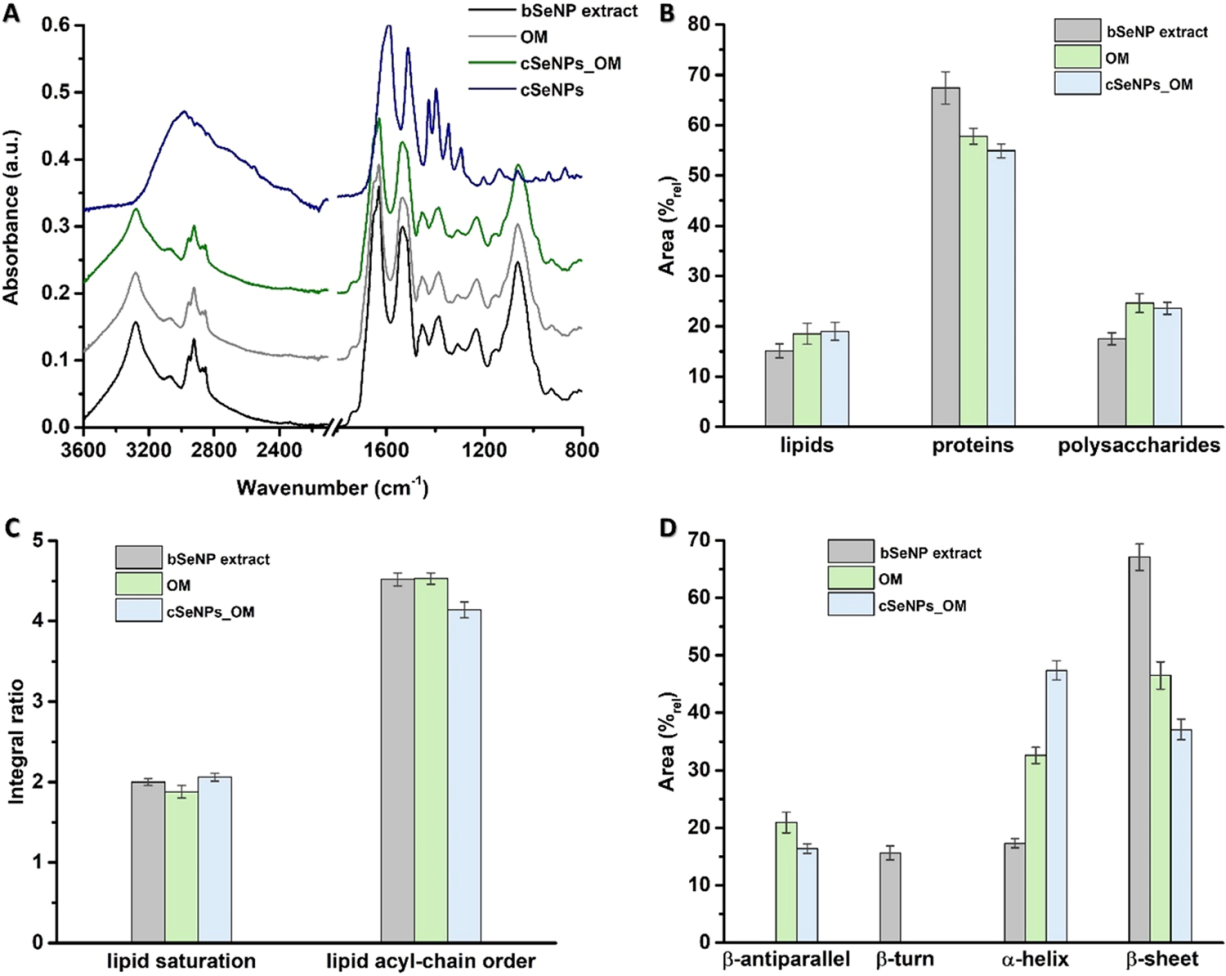
(A) ATR-FTIR spectra of the bSeNP extract, OM, cSeNPs, and cSeNPs_OM, (B) distribution of biomolecules (i.e., lipids, proteins, and polysaccharides), (C) degree of saturation and acyl-chain order, and (D) protein secondary structures based on ATR-FTIR analysis.

The nature of biomolecules within the bSeNP extract, OM, and cSeNPs_OM responsible for SeNP stabilization was evaluated through ATR-FTIR spectroscopy (Figure 2); the full band assignment of IR peaks is reported in Table S1.

Overall, ATR-FTIR spectra of the bSeNP extract, OM, and cSeNPs_OM exhibited vibrational modes typical of lipids, proteins, polysaccharides, and nucleic acids (Table S1), aligning with our previous characterization of the bSeNP extract recovered from the SeITE02 strain.^11,14,15,17−20,23^ Proteins, lipids, and polysaccharides make the OM amphiphilic, enabling its interaction and absorption onto the NP surfaces^24^ through electrostatic and steric interactions, a common feature among biogenic nanomaterials.^5,8,11,15,24,32,33^ Besides, part of the recovered OM seemed to assemble and organize in water, likely forming peculiar amphiphilic structures (Figure 1C), as highlighted in our earlier reports.^11,15^

ATR-FTIR spectroscopy also confirms the presence of these biomolecules within cSeNP_OM and validates the effectiveness of our incubation treatment for this sample. Indeed, IR signals of cSeNPs indicated the presence of cystine (RSSR), cystine monoxide (RSOSR), and cysteine sulfinic (RSO_2_^–^) functional groups, either as residues or adsorbed onto the SeNP surface (Table S1). These functional groups derived from the redox reaction between selenite and the amino acid l-cysteine,^24^ and, notably, they were absent, as maxima, in the cSeNPs_OM spectrum.

Vibrational modes and peak positions in ATR-FTIR spectra of biomolecule-containing samples displayed no obvious modification (Figure 2A and Table S1). Thus, to uncover potential differences between the spectra, integral calculations and spectral deconvolutions were performed (Figures 2B–D and S1; Tables S2 and S3). First, these spectral analyses revealed a significant variation in biomolecules’ abundance. Although proteins were present in all samples, the bSeNP extract showed the highest protein abundance (integral relative percentage). In contrast, the OM and cSeNPs_OM featured higher lipid and polysaccharide amounts than the former (Figure 2B). This observation supports the fundamental role of proteins in the generation, assembly, and stabilization of biogenic SeNPs, in line with previous reports.^8,10,14,15,19,24^

A deeper analysis of lipid IR contributions referring to −CH*_x_* stretching vibrations (2960–2850 cm^–1^) revealed comparable integral distribution for all of the samples (Figure S1), suggesting that these biomolecules did not experience sensible modification during the OM recovery from the bSeNP extract. The cSeNPs_OM sample exhibited a slight variation in these contributions, which may derive from a partial overlapping of −CH*_x_* signals from RSSR, RSOSR, and RSO_2_^–^ moieties (Table S1). The ratios between the asymmetric −CH_2_ stretching vibration (ca. 2925 cm^–1^) and either asymmetric −CH_3_ (ca. 2955 cm^–1^; *A*_νas(CH_2_)_/*A*_νas(CH_3_)_) or symmetric −CH_2_ (ca. 2850 cm^–1^; *A*_νas(CH_2_)_/*A*_νs(CH_2_)_) stretching vibrations indicate the fluidity and acyl chain disorder of lipids.^34^ Integral ratios for the OM were slightly lower than the bSeNP extract and cSeNPs_OM, whereas the latter showed the lowest degree of acyl-chain order (Figure 2C). These results suggest that the OM featured a lower amount of saturated lipids, these biomolecules were more disordered in cSeNPs_OM than in the other samples,^34^ and saturated lipids may preferentially interact with bSeNPs. In turn, lipids within the OM appear to have more freedom than in the bSeNP extract, making them able to organize and interact with other biomolecules. Furthermore, the variation of this integral ratio can relate to the increased number of −CH_3_ lipid groups within the OM, which might derive from the bSeNP removal, indicating the occurrence of interaction between lipid acyl chains and NPs. This hypothesis is further corroborated by the increased *A*_νas(CH_2_)_/*A*_νas(CH_3_)_ of cSeNPs_OM, which is comparable to that of the bSeNP extract (Figure 2C).

Spectral deconvolution also underlined differences in protein secondary structures between the samples (Figure S2A,C,E). The analysis of the amide I band enabled the identification of β-antiparallel (ca. 1680 cm^–1^), β-turn (ca. 1670 cm^–1^), α-helix (ca. 1650 cm^–1^), and β-sheet (1640–1610 cm^–1^) secondary structures differently distributed in the bSeNP extract, OM, and cSeNPs_OM (Table S2). Specifically, β-sheet secondary structures were the most abundant in the bSeNP extract and OM, whereas cSeNPs_OM displayed a higher content of α-helices than other samples (Figure 2D). The elevated β-sheet secondary structures of the bSeNP extract (Figure 2D) can trace back to Se-interacting and -reducing proteins or enzymes rich in cysteine residues, such as glutaredoxins, mycoredoxins, and/or the selenium-binding protein SeBP, which generally form β-strand structures, especially when interacting with selenite.^35−40^ The bSeNP extract also exhibited β-turn secondary structures (Figure 2D), which are fundamental for proper protein folding and stability, as they provide a directional change for the polypeptide chain through intramolecular hydrogen bonds.^41^ The biotransformation of selenite by *S. maltophilia* SeITE02 cells likely caused proteins either involved in the reduction process or interacting with SeNPs to form a tight network of hydrogen bonds and, hence, β-turn secondary structures. The absence of these latter structures, alongside the appearance of β-antiparallel protein contributions in ATR-FTIR spectra of OM and cSeNPs_OM (Figure 2D), suggests that the washing procedure of the bSeNP extract led to a partial protein unfolding, likely linked to the removal of SeNPs. The higher amount (relative percentage area) of α-helix secondary structures within the OM and cSeNPs_OM than the bSeNP extract (Figure 2D) indicated the propensity of β-sheet-containing proteins to interact with bSeNPs through hydrogen bonds, van der Waals interactions, or L- and X-type ligand interactions via their −NH_2_ and −COO^–^ moieties,^24^ remaining anchored to Se atoms on the NP surface. Specifically, the elevated contribution deriving from α-helix secondary structures within cSeNPs_OM may depend on (i) the interaction of proteins with RSSR, RSOSR, and RSO_2_^–^ residues and their re-organization or (ii) a partial protein unfolding caused by cSeNPs.

### Susceptibility of *S. cerevisiae* to bSeNP Extract, cSeNPs, cSeNPs_OM, or OM

One of the most interesting applicative properties of the bSeNP extract deriving from SeITE02 cells is its antimicrobial activity against both planktonic cells and biofilms of diverse Gram-negative and -positive pathogens, including some clinical isolates.^17,18,23^ This feature links directly to the OM and its integrity, as cSeNPs having similar size and morphologies as those biogenic did not efficaciously inhibit pathogen growth.^18,20,23^ Thus, bSeNP extracts may play a vital role in fighting pathogen infections, which, due to the exponential spreading of antimicrobial resistance, is an enormous biotechnological value of these formulations.

**Figure 3:**
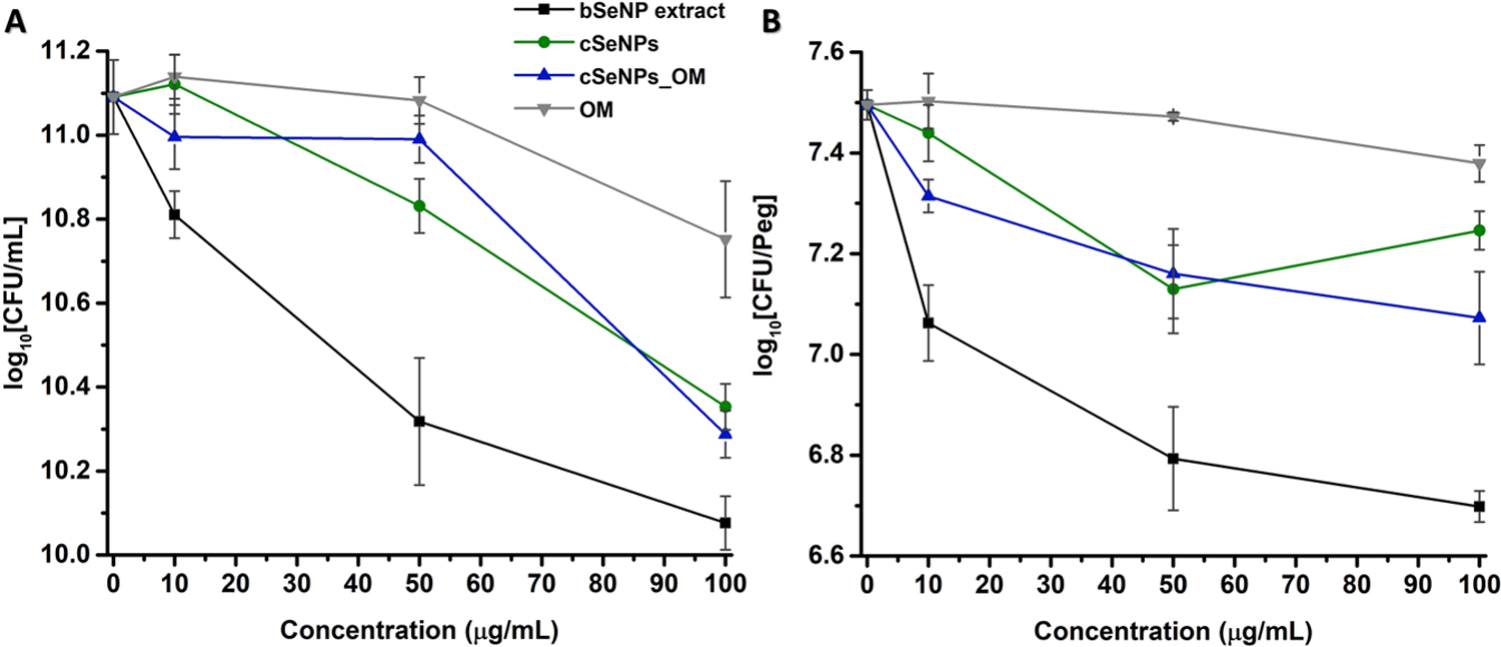
Comparative susceptibility curves of SeNPs-containing or -deriving formulations to *S. cerevisiae* cells grown (A) planktonically or (B) as a biofilm.

In the present study, the yeast *S. cerevisiae* was used as a eukaryotic model to study its susceptibility, as planktonic cells or a biofilm, against bSeNP extract, cSeNPs, and cSeNPs_OM (Figure 3).

Overall, none of the tested formulations efficiently inhibited *S. cerevisiae* growth, although the bSeNP extract showed slightly more of an effect than the cSeNPs, cSeNPs_OM, or OM, determining a 1 or 0.5 logarithm decrease in the CFU/mL of yeast planktonic cells or biofilm (Figure 3), respectively. On the contrary, the sole OM did not affect the yeast growth, suggesting that the modest bSeNP extract activity derived from a synergic effect exerted by the combination of SeNPs and the OM (Figure 3). Indeed, cSeNPs_OM showed an intermediate efficacy between cSeNPs and the bSeNP extract against yeast planktonic cells, yet no difference in activity with chemogenic NPs was observed in the case of the biofilm (Figure 3). This result agrees with the cytocompatibility of similar formulations assessed by Cremonini and colleagues^18^ against dendritic cells and fibroblasts and further sustains their potential in the biomedical field. Nevertheless, a deeper understanding of the interaction(s) between these biogenic formulations and eukaryotic systems is required to assess better the applicability of bSeNP extracts.

### Interaction of bSeNP Extract, cSeNPs, OM, and cSeNPs_OM with Eukaryotic Model Membranes

The primary target of any substance or material interfacing with a cell is the membrane, a dynamic ensemble of macromolecules that constitute the first defence against any threats. Yet, given the complexity of the cell membrane, several simpler membrane-like models have been developed to evaluate the biological impact of innovative materials or formulations, such as polar lipid extracts and phospholipid vesicles. Here, LUVs prepared from *S. cerevisiae* polar lipid extract (YE lipid; *d*_H_ = 115 ± 3 nm; ζ-potential = −30.3 ± 1.2 mV; Figure S3), or its most represented lipids—i.e., the zwitterionic POPC (*d*_H_ = 145 ± 2 nm; ζ-potential = −5.30 ± 1.37 mV) and DMPC (*d*_H_ = 144 ± 1 nm; ζ-potential = −3.48 ± 0.39 mV), were used to investigate the effect of the bSeNP extract, cSeNPs, OM, and cSeNPs_OM on the fluidity of a eukaryotic membrane focusing on both the interface (headgroups) region and the hydrophobic core of the lipid bilayer. Specifically, the role of the acyl chain architecture in the lipid–SeNP sample interactions was evaluated by using the monosaturated POPC and the fully saturated DMPC.

The NP–liposome interaction depends on the gel–liquid-crystalline equilibrium of the lipid vesicles. At temperatures higher than the melting temperature (*T*_m_), lipids in the vesicles are in a fluid liquid-crystalline state, where they can diffuse more freely and are more exposed to contact NPs.^42^ When investigating the NP–lipid vesicle interaction, the overall system to consider comprises these two elements alongside the ligands/capping agents on the NP surface. In this system, multiple parameters (e.g., the charge of each component, the NP size, and the nature of both the ligands/capping agents and the chosen lipids) affect the NP–vesicle interaction.^42^ Thus, in the next subsections, we first report results regarding fluidity variations of both interphase (headgroups) regions and hydrophobic core of LUVs upon their incubation with the bSeNP extract, cSeNPs, OM, and cSeNPs_OM, and, subsequently, we discuss our findings considering all of the mentioned parameters influencing the sample–liposome interaction.

#### Fluidity Variations in the Interphase and Headgroup Regions of LUVs

**Figure 4:**
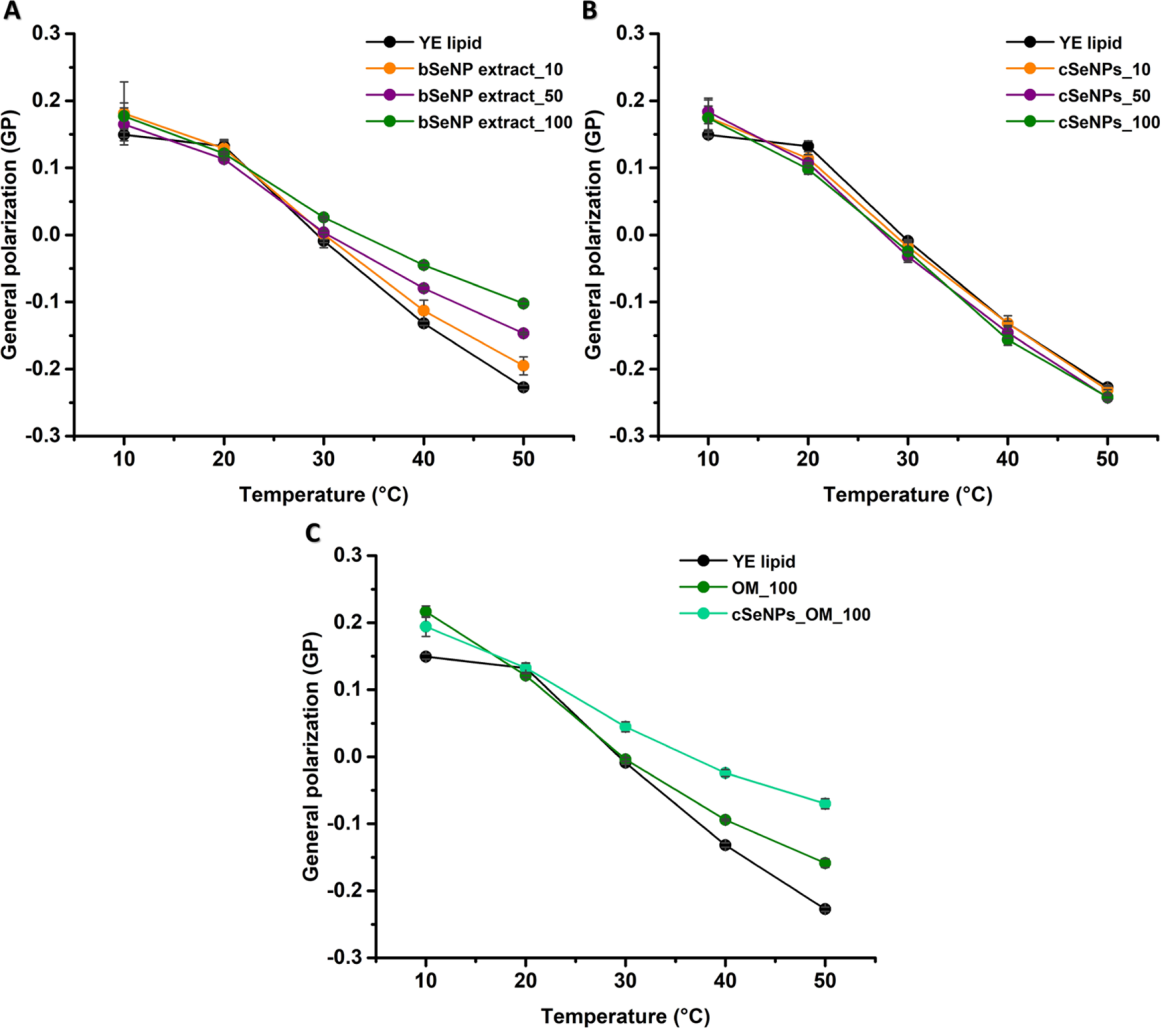
Generalized polarization (GP) values of laurdan in yeast polar extract vesicles (YE lipid) incubated with (A) the bSeNP extract (10, 50, or 100 μg/mL), (B) cSeNPs (10, 50, or 100 μg/mL), and (C) the OM (100 μg/mL) or cSeNPs_OM (100 μg/mL). Data are plotted as an average of three replicates ± standard deviation.

The potential impact of biogenic or chemogenic SeNPs on a model eukaryotic membrane was investigated first on YE lipid LUVs focusing on variations of GP as a function of the temperature, which reflect the change of vesicle fluidity in the presence of various challenges. The LUVs contain a mixture of lipids and have a *T*_m_ of ∼20 °C. The initial GP value of 0.149 ± 0.004 reflects a more rigid gel phase, which drops at temperatures above 20 °C, indicating increased fluidity with increased temperature (Figure 4).

The dose-dependent addition of cSeNPs exhibited negligible effects on the fluidity and melting curve of the YE lipid vesicles (Figure 4B), while the bSeNP extract had a large impact (Figure 4A). Indeed, the bSeNP dose-dependent response induced significant rigidification of these bilayers, as indicated by GP values from −0.027 ± 0.003 at the lowest concentration to +0.022 ± 0.004 at the highest concentration at the relevant physiological temperature of 30 °C. The addition of cSeNPs_OM_100 increases GP values partially resembling the curves of the bSeNP extract (Figure 4A,C), indicating rigidification of the membranes. The OM, tested at its highest concentration (100 μg/mL), only slightly rigidified the yeast LUVs at temperatures higher than 30 °C (Figure 3C).

**Figure 5:**
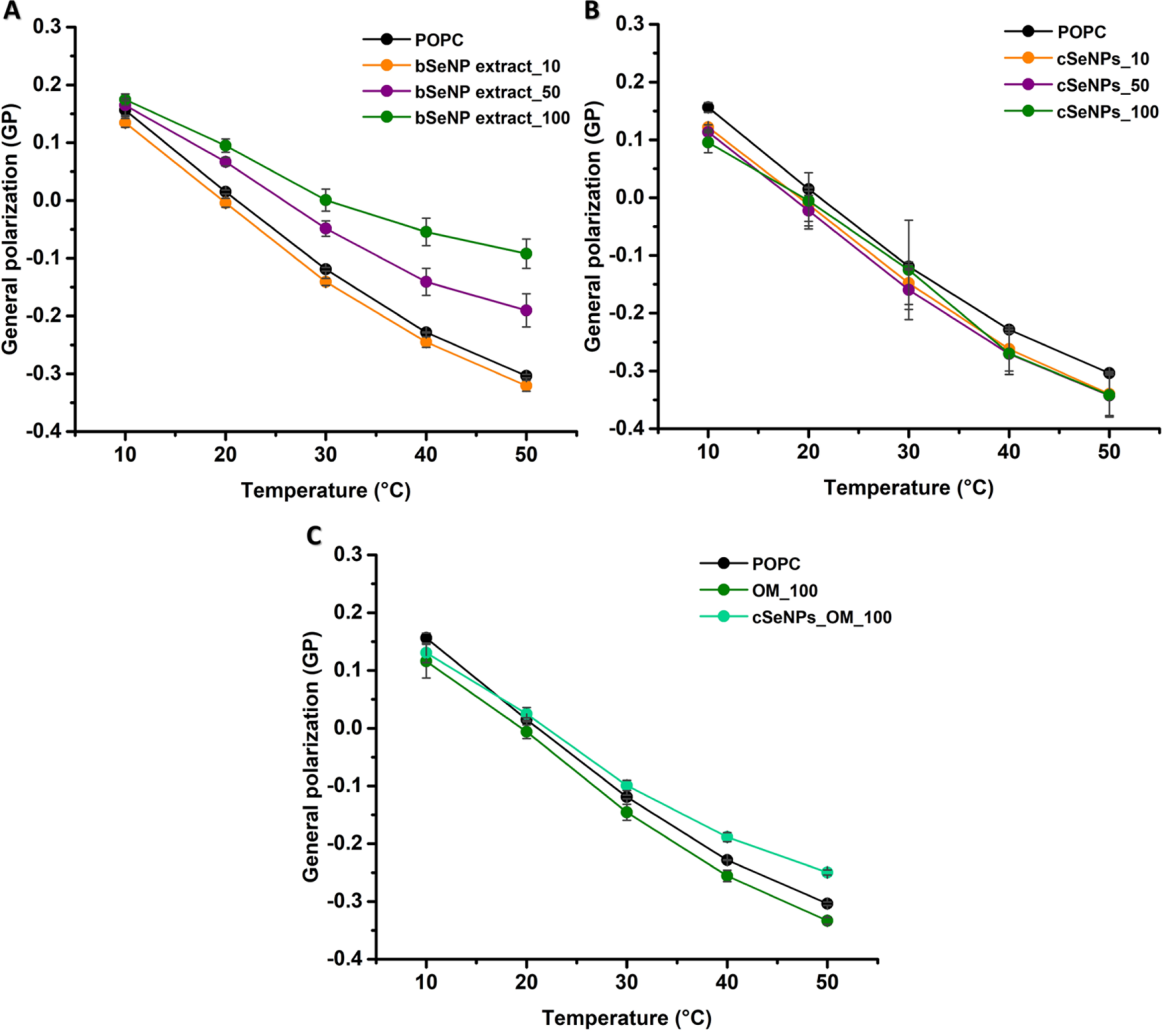
Generalized polarization (GP) values of laurdan in POPC vesicles incubated with (A) the bSeNP extract (10, 50, or 100 μg/mL), (B) cSeNPs (10, 50, or 100 μg/mL), and (C) the OM (100 μg/mL) or cSeNPs_OM (100 μg/mL). Data are plotted as an average of three replicates ± standard deviation.

Subsequently, our investigation focused on LUVs prepared from the eukaryotic lipid models POPC and DMPC. The monounsaturated POPC has a *T*_m_ below 0 °C;^43^ the unexposed POPC vesicles showed a continuous decrease of GP, indicating a progressively more fluid membrane with increasing temperature (Figure 5).

Focusing on 30 °C, POPC had a GP value of −0.119 ± 0.008, which increased by 11-fold at the largest bSeNP concentration tested, indicating a high rigidification of these LUVs (Figure 5A). In contrast, adding cSeNPs did not result in statistically significant GP variations of POPC vesicles, while the OM_100 led to a slight fluidification (decrease up to 1.3-fold in the GP values), which was observed throughout the tested temperature range (Figure 5B,C). Finally, cSeNPs_OM_100 moderately rigidified the POPC vesicles, as proved by the shifting up of their GP curve (Figure 5C). The latter result suggests that the OM can influence the SeNP–lipid interaction favoring lipid vesicle rigidification.

**Figure 6:**
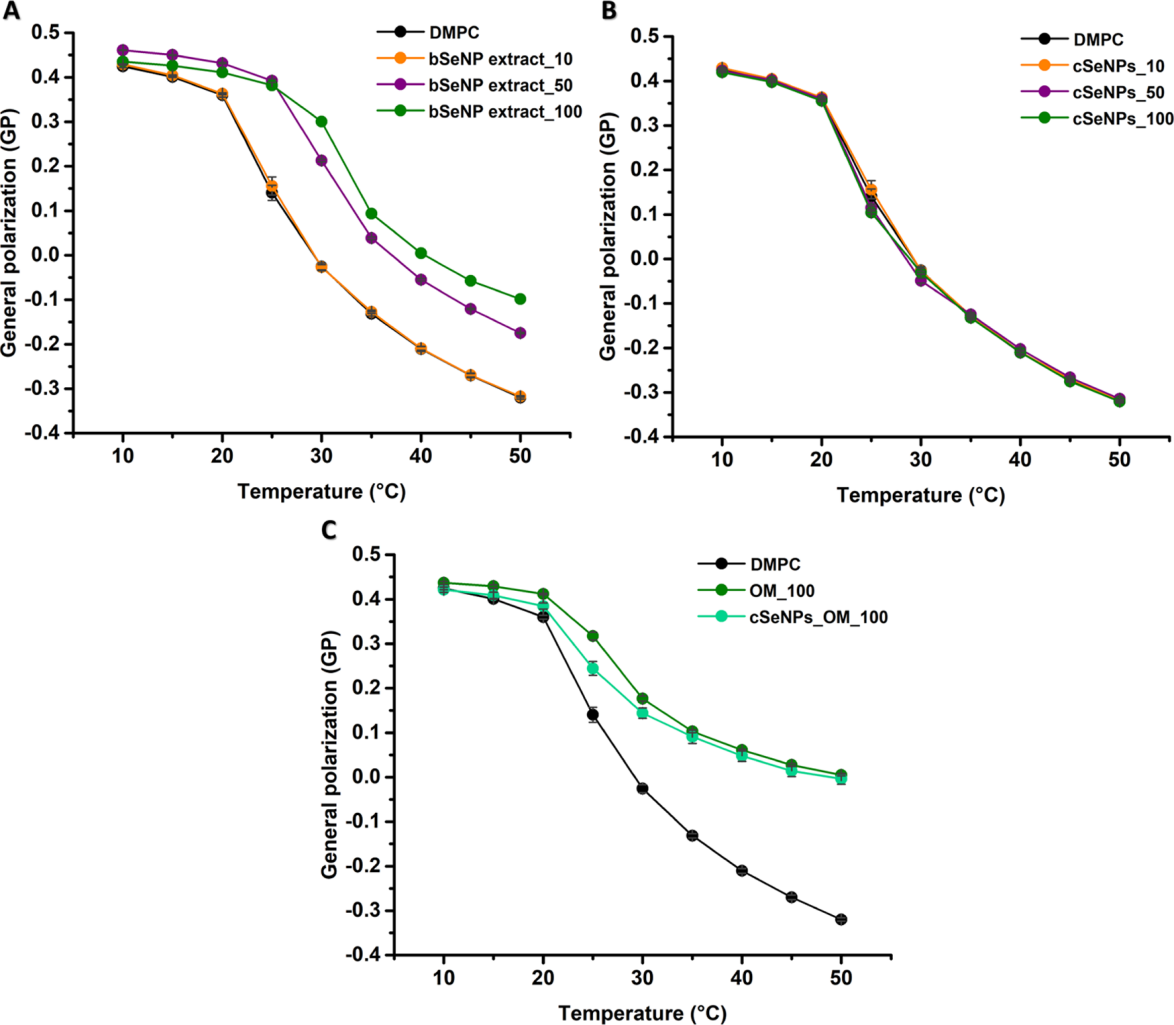
Generalized polarization (GP) values of laurdan in DMPC vesicles incubated with (A) the bSeNP extract (10, 50, or 100 μg/mL), (B) cSeNPs (10, 50, or 100 μg/mL), and (C) the OM (100 μg/mL) or cSeNPs_OM (100 μg/mL). Data are plotted as an average of three replicates ± standard deviation.

In the case of DMPC vesicles, the initial GP value of 0.425 ± 0.003 reflects tightly packed membranes at 10 °C (Figure 6), as expected from a fully saturated lipid in the gel phase. The GP curve starts with high readings reflecting a rigid gel phase but drops in an inverse sigmoidal shape upon heating (Figure 6). The midpoint of the inflection corresponds to the melting temperature, whereas lower readings at higher temperatures reflect a fluid liquid-crystalline phase. DMPC LUVs exhibited an inflection point at 25 °C, which relates well to an expected *T*_m_ of ca. 24 °C.^28^

Upon DMPC exposure to cSeNPs, no changes in GP values were detected (Figure 6B). Treatments of DMPC vesicles with bSeNP extract resulted in fluidity changes, including a shift of the onset of melting from 20 to almost 30 °C with higher amounts for the addition of bSeNP extract (Figure 6A). Moreover, the OM_100 greatly influenced DMPC vesicle fluidity, by inducing a strong rigidification of LUVs (Figure 6C). Similar results were obtained for cSeNPs_OM_100 (Figure 6C), reflecting that OM can influence the fluidity of the fully saturated DMPC vesicles without relevant changes in their melting temperature.

#### Fluidity Variations in Model Lipid Vesicles’ Hydrophobic Core

**Figure 7:**
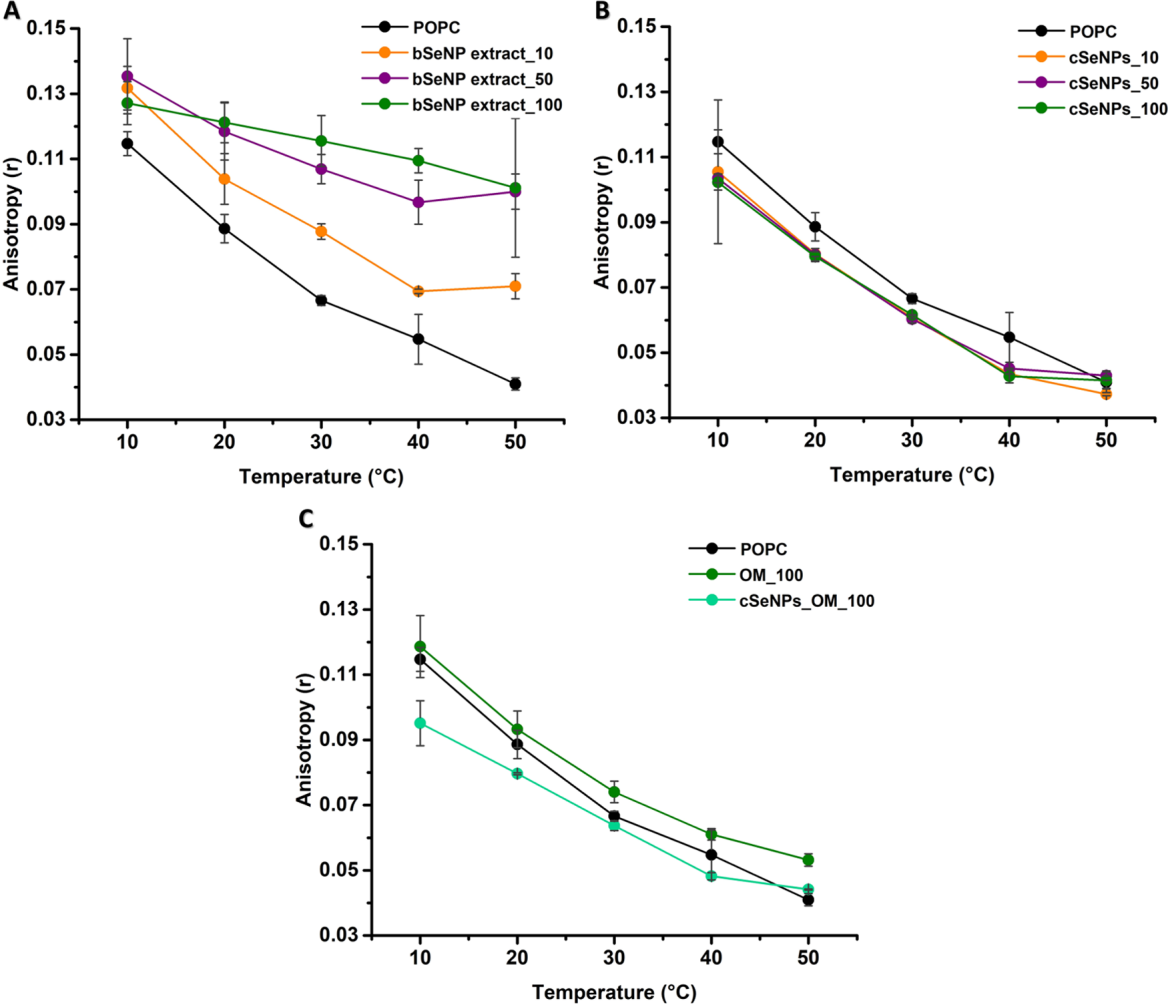
Fluorescence anisotropy values of DPH in POPC vesicles incubated with (A) the bSeNP extract (10, 50, or 100 μg/mL), (B) cSeNPs (10, 50, or 100 μg/mL), and (C) the OM (100 μg/mL) or cSeNPs_OM (100 μg/mL). Data are plotted as an average of three replicates ± standard deviation.

**Figure 8:**
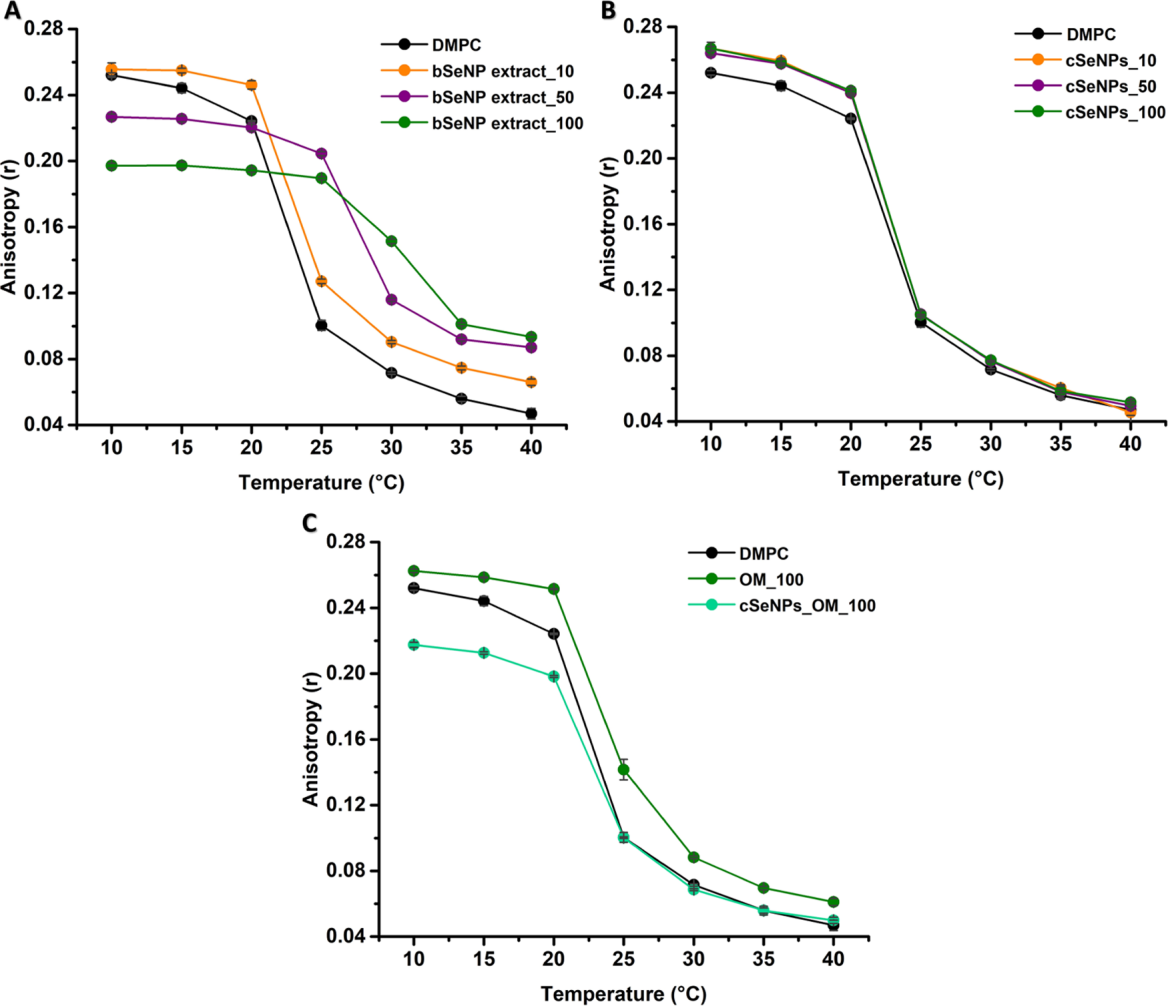
Fluorescence anisotropy values of DPH in DMPC vesicles incubated with (A) the bSeNP extract (10, 50, or 100 μg/mL), (B) cSeNPs (10, 50, or 100 μg/mL), and (C) the OM (100 μg/mL) or cSeNPs_OM (100 μg/mL). Data are plotted as an average of three replicates ± standard deviation.

To further verify the differences between cSeNPs and bSeNP extract interacting with lipid LUVs, DPH anisotropy ⟨*r*⟩ was employed to assess their fatty acyl tail dynamics (Figures 7 and 8). While the *T*_m_ for POPC is below 0 °C, any NP-induced rigidification extending into the hydrophobic core would be detected.

The bSeNP extract led to a great increase in ⟨*r*⟩ values (Figure 7A), which further highlights its rigidification effect on POPC LUVs in a concentration-dependent fashion, consistently with GP results (Figure 3A). cSeNPs determined a minor statistically significant decrease in POPC anisotropy at 30 °C (Figure 7B), which indicates a low fluidizing effect. The incubation of POPC LUVs with the OM_100 led to a moderate increase in order within the hydrophobic core, while cSeNPs_OM_100 mediated a slight fluidification of the lipid vesicles at 10 °C (Figure 7C).

The effects of SeNPs and the OM on the fluidity of the DMPC hydrophobic core are illustrated in Figure 8.

DMPC LUVs exhibited ⟨*r*⟩ values of 0.252 ± 0.001 in the gel phase (10 °C), which changed to 0.056 ± 0.002 at 35 °C (Figure 8) after melting into the liquid-crystalline phase. These vesicles featured a *T*_m_ of 23 °C, close to the expected melting temperature of 24 °C.^43^ The bSeNP extract causes changes in rigidity in both the gel and liquid-crystalline phases of DMPC vesicles (Figure 8A). Similar results were also observed upon DMPC vesicle incubation with the sole OM (Figure 8C). Additionally, increasing the bSeNP extract concentration caused a shift in the melting temperature from 24 to 32 °C in a dose-dependent manner (Figure 8A). In the case of cSeNPs, no effect on DMPC lipid mobility in the gel phase (<20 °C) and no significant variation of their *T*_m_ was observed (Figure 8B). Lastly, cSeNPs_OM decreased ⟨*r*⟩ values in the gel phase (10 °C) but had no effect in the liquid-crystalline one (Figure 8C).

#### Effect of SeNPs on Model Membranes

The NP surface charge determines electrostatic and van der Waals contributions responsible for the NP adhesion to the lipid interface.^42^ The SeNP-containing samples and LUVs tested in the present study featured negative ζ-potential values (Table 1 and Figure S3B). Thus, electrostatic repulsions were likely to develop between these two elements, which may have limited the NP adhesion onto the lipid vesicles. Indeed, theoretical studies showed that hydrophilic NPs with a high surface charge, which, in our context, would describe cSeNPs surrounded and stabilized by RSSR, RSOSR, and RSO_2_^–^ residues, reside on the surface of a lipid bilayer.^44^ Besides, NPs with a diameter larger than the so-called critical NP diameter (*d*_c_; minimum size for the spontaneous wrapping of NPs) will be engulfed in the lipid bilayer.^45−47^ The *d*_c_ depends on the nature of the NP core and surface. Indeed, the *d*_c_ of silica (SiO_2_) NPs varies between 20 and 30 nm,^48,49^ while that of gold NPs (AuNPs) is ca. 4 nm.^50^ Considering the size of SeNPs (40 nm < *d*_avg_ < 50 nm) within the tested samples, a portion of these NPs should be internalized within lipid vesicles without causing either rupture or modification of the lipid bilayer.^42,47−50^ Moreover, Contini and colleagues^47^ determined a partial wrapping of some large (25–50 nm) AuNPs on the outer layer of the lipid membrane, as only a small number of these sized NPs is required to cause the filling of 180 nm liposomes. The same authors also detected several large free AuNPs not interacting with liposomes. Thus, the absence of fluidity changes upon LUVs incubation with different concentrations of cSeNPs (Figures 4–8) may link to (i) repulsive electrostatic interactions, as these NPs have strong negative ζ-potential values, (ii) their total or partial adsorption onto lipid vesicles, and/or (iii) their inability to adhere onto the LUV surface likely due to the inaccessibility of the lipid vesicles to the NP entry.

#### Effect of the Organic Material on Model Membranes

Although similar considerations on the charge and size of SeNPs can be made for LUVs facing the bSeNP extract and cSeNPs_OM, these samples showed to overall decrease the vesicle fluidity, mediating their rigidification (Figures 4–8). This evidence, in addition to the more modest rigidification effect observed for lipid vesicles incubated with the OM (Figures 4–8), underlines the pronounced influence played by ligands/capping agents surrounding the NP surface in the interactions between NPs and lipid membranes.^42,51,52^ Specifically, the higher effect observed for rigidification and *T*_m_ variation in DMPC than POPC vesicles may derive from the higher saturation degree of the former, which leads to generating more ordered structures with an increased membrane bending rigidity.^47^ Biomolecules within the OM can interact with lipid vesicles through polar headgroups and/or the hydrophobic chains of lipids. In both cases, this event will influence the lipid packing inducing modification in the vesicle structure and thermotropic behavior.^42^ In this regard, several studies reported that sugars (e.g., trehalose, sucrose, and oligosaccharides) and proteins cause a change in lipid fluidity, inducing a rigidification of vesicles.^50,51,53−58^ Sugars can simultaneously interact with multiple lipid molecules in the fully hydrated liquid-crystalline state, mostly bridging through hydrogen bonds with the oxygens of the phosphate (head) groups of zwitterionic phospholipids.^59^ This event leads to the sugar adsorption to the lipid bilayer interface, increasing the surface concentration of these biomolecules onto LUVs.^53^ As a result, a hydration layer is formed on the LUV surface, which decreases the lateral mobility of lipids in vesicles, protecting them from rupture or leakage events.^51^ In turn, the limited lipid lateral mobility determines LUV rigidification, which is in line with our observations (Figures 3–7). This phenomenon depends on the sugar concentration and length and is more pronounced for long oligosaccharides.^53^ Polysaccharides, an important component of OM-containing samples (Figure 2A,B and Table S1), were also able to rigidify lipid bilayers by (i) generating a high number of hydrogen bonds, (ii) developing electrostatic interactions, and (iii) increasing the compactness of lipid inner chains.^60,61^ In the case of proteins, complex ensembles of these biomolecules, such as those in the OM (Figures 2A,D and S2, and Table S2), adsorb onto the lipid vesicle surface, forming a rigid and adhesive protein corona.^62,63^ Depending on protein steric hindrance, amphiphilic nature, specific functional groups, and charge, this protein corona can develop hydrophobic, electrostatic, or hydrogen bond interactions with lipids,^52,62^ which modify the lipid vesicle fluidity. The latter often corresponds to a rigidification effect and the simultaneous existence of both the gel and liquid-crystalline lipid states,^54−58^ further corroborating the results presented here for samples containing the OM (Figures 4–8). Moreover, the protein corona can increase the thickness of the lipid bilayer, mediate the adsorption of a second protein layer (i.e., outer corona), and modulate the vesicle diameter.^63^ For instance, Wolfram and colleagues^62^ indicated that proteins impermeable to LUVs cause a variation in their osmotic pressure, leading to the loss of water molecules from the hydrophobic core of vesicles, determining a shrinkage in their size. Another important class of biomolecules within the OM is lipids, which, depending on their nature, will partition the vesicles’ lipid bilayer. SeITE02 membrane lipids can develop hydrogen bonds and steric interactions with the LUV bilayer in the fluid state. In the latter, hydrophobic chains and hydrocarbon groups of LUV lipids are partially exposed to the aqueous phase,^52^ making their contact with other molecules more likely. Consequently, the insertion of SeITE02 lipids in LUVs may modify the properties of lipids within LUVs, such as their *T*_m_, type of phases, domains, fluidity, and permeability. In this regard, hydrophobic ligands of AuNPs were reported to increase the *T*_m_ of dipalmitoylphosphatidylcholine/dipalmitoylphosphatidylglycerol vesicles rigidifying them.^64^ The nature of the lipids produced by SeITE02 cells during selenite incubation, and inherited by the bSeNP extract, likely governs the interaction of these biomolecules with LUVs. In this regard, bacteria can increase the saturation degree of fatty acids to counteract metal(loid) toxicity, modulating the membrane fluidity.^40,65^ Furthermore, *S. maltophilia* strains appear to naturally contain more saturated fatty acids than unsaturated ones.^66^ Thus, saturated fatty acids of SeITE02 cells might play a pivotal role in the rigidification of LUVs incubated with the bSeNP extract, OM, or cSeNPs_OM (Figures 4–8).

#### Comparison between the Effect of Organic Material-Containing Suspensions on Model Membranes

The OM, bSeNP extract, and cSeNPs_OM caused an overall rigidification of LUVs, yet some differences in their effect were observed (Figures 4–8). For instance, the GP variation related to rigidified POPC LUVs followed the order bSeNP extract > cSeNPs_OM_100 > OM, while DMPC vesicles showed an opposite trend (Figures 5 and 6). This outcome may derive from (i) the diverse biomolecules within the bSeNP extract and the OM and (ii) the saturation degree of these LUVs. Indeed, ATR-FTIR spectroscopy revealed that lipids and polysaccharides are represented the most in the OM and cSeNPs_OM, while proteins were the most abundant for the bSeNP extract (Figure 2B). This outcome aligns with our previous study on the biogenic SeNP extract, OM, and washed biogenic extract obtained from *Micrococcus* sp. cells^24^ and indicates that the OM featured a higher percentage of hydrophobic (or partially hydrophobic) biomolecules than the original biogenic extract. Moreover, POPC LUVs in a liquid-crystalline state are more fluid than DMPC ones, as the former contains one unsaturation. This feature, in turn, should determine more inclusions of water molecules in POPC vesicles than DMPC at a temperature greater than *T*_m_. Indeed, the latter showed a higher overall rigidity than POPC vesicles without adding any challenges (Figures 4–5). As a result, hydrophobic interactions should influence more GP changes in DMPC LUVs, which is in line with the high rigidification effect mediated in these vesicles by the OM and cSeNPs_OM_100 (Figure 5a,c). Besides, differences observed through ATR-FTIR spectroscopy for biomolecule abundance, protein secondary structures, and lipid order and fluidity (Figure 2) suggest that SeNPs within the bSeNP extract and cSeNPs_OM may act as a hot spot for the OM wrapping around the NPs themselves, increasing the local concentration of biomolecules interacting with LUVs. This phenomenon, alongside the more hydrophilic nature of the bSeNP extract and cSeNPs_OM than the OM, can explain the greater rigidification of YE lipid and POPC vesicles upon incubation with the former ones (Figures 4A,C and 5A,C). A similar conclusion can be drawn by analyzing the hydrophobic core of POPC LUVs, for which the highest rigidification effect was detected when adding the bSeNP extract to the vesicles (Figure 7A). In the case of DMPC vesicles, anisotropy experiments revealed the highest effect for the bSeNP extract, followed by the OM_100 (Figure 8A,C). Moreover, SeNP presence fluidized at first (temperature <20 °C) these lipid vesicles (Figure 8A,C), which may trace back to the partial or total wrapping of large SeNPs (*d*_avg_ = 50 nm) in LUVs.^47^ This perturbation may have caused modifications in the vesicle bilayer, its elastic properties, and curvature.^42^ Since DMPC is more rigid than POPC, these modifications might alter to a greater extent the former, which, to avoid collapsing or fusion, can adopt a more fluid conformation re-orienting, for instance, its acyl chain. Once the temperature reached its *T*_m_, biomolecules within the bSeNP extract may have interacted more easily with DMPC acyl chains, amplifying the rigidification effect on the vesicle hydrophobic core.

## Conclusions

The present study investigates the cytocompatibility of the biogenic SeNP extract recovered from *S. maltophilia* SeITE02 cells or chemogenic SeNPs on the eukaryotic model *S. cerevisiae* focusing on their interaction with the headgroups and hydrophobic core of vesicles obtained from the yeast polar lipid extract or defined lipids with different saturation degrees (i.e., POPC and DMPC) mimicking the cell membrane. Moreover, since our previous studies demonstrated that the biogenic SeNP extract contains organic material, we investigated fluidity changes occurring upon vesicle incubation with this material or chemogenic SeNPs exposed to it. Our results showed that the biogenic SeNP extract, the organic material, and chemogenic SeNPs incubated with it induced rigidification of lipid vesicles, which was enhanced for the former, while chemogenic SeNPs alone did not cause significant modifications. Given the similarities, in terms of size and shape, between biogenic and chemogenic SeNPs, this outcome highlighted the organic material’s importance in modulating the lipid bilayer properties through the occurrence of interactions between its biomolecules and lipid vesicles and, in turn, in governing physical–chemical and biological properties of the biogenic SeNP extract. The in vitro vesicle rigidification effect promoted by interacting biogenic SeNP extract could reflect, in vivo, a sort of protective mechanism against the same stressor, confirming its low toxicity toward *S. cerevisiae* cells. This evidence adds novel information on the suitability of such biogenic nanomaterial concerning its biocompatibility, thus supporting their potential application as antimicrobials avoiding harmful side effects for the host.

## Supporting Information Available

ATR-FTIR absorption bands and identification of the bSeNP extract, cSeNPs, OM, and cSeNPs_OM (Table S1); integral distribution of lipid −CH*_x_* stretching vibrations obtained for the analyzed samples from ATR-FTIR spectra (Figure S1); deconvolution of ATR-FTIR spectra of (A, B) bSeNP extract, (C, D) OM, and (E, F) cSeNPs_OM in the (A, C, E) 1780–1420 and (B, D, F) 1420–940 cm^–1^ regions (Figure S2); identification of IR absorption bands obtained through deconvolution of ATR-FTIR spectra of the bSeNP extract, cSeNPs, OM, and cSeNPs_OM in the 1780–1420 cm^–1^ region (Table S2); identification of IR absorption bands obtained through deconvolution of ATR-FTIR spectra of the bSeNP extract, cSeNPs, OM, and cSeNPs_OM in the 1420–950 cm^–1^ region (Table S3); (A) hydrodynamic diameter and (B) ζ-potential values measured for yeast extract, POPC, and DMPC LUVs (Figure S3) (PDF)

## References

[ref1] Rao C. N. R.; Muller A.; Cheetham A. K. The Chemistry of Nanomaterials: Synthesis, Properties and Applications; WILEY-VCH Verlag GmbH & Co. KgaA: Weinheim, Germany, 2004; pp 1–11.

[ref2] Talapin D. V.; Lee J. S.; Kovalenko M. V.; Shevchenko E. V. Prospects of colloidal nanocrystals for electronic and optoelectronic applications. Chem. Rev. 2010, 110, 389–458. 10.1021/cr900137k. 19958036

[ref3] Abdussalam-Mohammed W. Comparison of chemical and biological properties of metal nanoparticles (Au, Ag) with metal oxide nanoparticles (ZnO-NPs) and their applications. Adv. J. Chem., Sect. A 2020, 3, 192–210. 10.33945/SAMI/AJCA.2020.2.8.

[ref4] Piacenza E.; Presentato A.; Zonaro E.; Lampis S.; Vallini G.; Turner R. J. Selenium and Tellurium Nanomaterials. Phys. Sci. Rev. 2018, 3, 20170100 10.1515/psr-2017-0100.

[ref5] Piacenza E.; Presentato A.; Turner R. J. Stability of biogenic metal(loid) nanomaterials related to the colloidal stabilization theory of chemical nanostructures. Crit. Rev. Biotechnol. 2018, 38, 1137–1156. 10.1080/07388551.2018.1440525. 29480081

[ref6] Grasso G.; Zane D.; Dragone R. Microbial nanotechnology: challenges and prospects for green biocatalytic synthesis of nanoscale materials for sensoristic and biomedical applications. Nanomaterials 2020, 10, 11 10.3390/nano10010011. PMC7023511 31861471

[ref7] Ilyas S.; Kim M. S.; Lee J. C.; Jabeen A.; Bhatti H. N. Bio-Reclamation of Strategic and Energy Critical Metals from Secondary Resources. Metals 2017, 7, 207 10.3390/met7060207.

[ref8] Shoeibi S.; Mozdziak P.; Golkar-Narneji G. Biogenesis of selenium nanoparticles using green chemistry. Top. Curr. Chem. 2017, 375, 88 10.1007/s41061-017-0176-x. 29124492

[ref9] Ruiz-Fresneda M. A.; Staicu L. C.; Lazuen-Lopez G.; Merroun M. L. Allotropy of selenium nanoparticles: colourful transition, synthesis, and biotechnological applications. Microb. Biotechnol. 2023, 16, 877–892. 10.1111/1751-7915.14209. 36622050 PMC10128136

[ref10] Sarkar J.; Mridha D.; Davoodbasha M. A.; Banerjee J.; Chanda S.; Ray K.; Roychowdhury T.; Acahrya K.; Sarkar J. A state-of-the-art systemic review on Selenium nanoparticles: mechanisms and factors influencing biogenesis and its potential applications. Biol. Trace Elem. Res. 2023, 1–37. 10.1007/s12011-022-03549-0. 36633786

[ref11] Piacenza E.; Presentato A.; Heyne B.; Turner R. J. Tunable photoluminescence properties of selenium nanoparticles: biogenic versus chemogenic synthesis. Nanophotonics 2020, 9, 3615–3628. 10.1515/nanoph-2020-0239.

[ref12] Sanchez S. A.; Tricerri M. A.; Gratton E. Laurdan generalized polarization fluctuations measures membrane packing micro-heterogeneity in vivo. Proc Natl. Acad. Sci. U.S.A. 2012, 109, 7314–7319. 10.1073/pnas.1118288109. 22529342 PMC3358851

[ref13] Gidwani A.; Holowka D.; Baird B. Fluorescence anisotropy measurements of lipid order in plasma membranes and lipid rafts from RBL-2H3 mast cells. Biochemistry 2001, 40, 12422–12429. 10.1021/bi010496c. 11591163

[ref14] Lampis S.; Zonaro E.; Bertolini C.; Cecconi D.; Monti F.; Micaroni M.; Turner R. J.; Butler C. S.; Vallini G. Selenite biotransformation and detoxification by *Stenotrophomonas maltophilia* SeITE02: Novel clues on the route to bacterial biogenesis of selenium nanoparticles. J. Hazard. Mater. 2017, 324, 3–14. 10.1016/j.jhazmat.2016.02.035. 26952084

[ref15] Piacenza E.; Presentato A.; Ambrosi E.; Speghini A.; Turner R. J.; Vallini G.; Lampis S. Physical-chemical properties of biogenic selenium nanostructures biosynthesis by two environmental isolates *Stenotrophomonas maltophilia* SeITE02 and *Ochrobactrum* sp. MPV1. Front. Microbiol. 2018, 9, 3178 10.3389/fmicb.2018.03178. 30619230 PMC6306038

[ref16] Li Q.; Chen T.; Yang F.; Liu J.; Zheng W. Facile and controllable one-step fabrication of selenium nanoparticles assisted by L-cysteine. Mater. Lett. 2010, 64, 614–617. 10.1016/j.matlet.2009.12.019.

[ref17] Zonaro E.; Lampis S.; Turner R. J.; Qazi S. J. S.; Vallini G. Biogenic selenium and tellurium nanoparticles synthetized by environmental microbial isolates efficaciously inhibit bacterial planktonic cultures and biofilms. Front. Microbiol. 2015, 6, 584 10.3389/fmicb.2015.00584. 26136728 PMC4468835

[ref18] Cremonini E.; Zonaro E.; Donini M.; Lampis S.; Boaretti M.; Disu S.; Melotti P.; Lleo M. M.; Vallini G. Biogenic selenium nanoparticles: characterization, antimicrobial activity and effects on human dendritic cells and fibroblasts. Microb. Biotechnol. 2016, 9, 758–771. 10.1111/1751-7915.12374. 27319803 PMC5072192

[ref19] Bulgarini A.; Lampis S.; Turner R. J.; Vallini G. Biomolecular composition of capping layer and stability of biogenic selenium nanoparticles synthesized by five bacterial species. Microb. Biotechnol. 2021, 14, 198–212. 10.1111/1751-7915.13666. 33068075 PMC7888468

[ref20] Piacenza E.; Presentato A.; Zonaro E.; Lemire J. A.; Demeter M.; Vallini G.; Turner R. J.; Lampis S. Antimicrobial activity of biogenically produced spherical Se-nanomaterials embedded in organic material against *Pseudomonas aeruginosa* and *Staphylococcus aureus* strains on hydroxyapatite-coated surfaces. Microb. Biotechnol. 2017, 10, 804–818. 10.1111/1751-7915.12700. 28233476 PMC5481514

[ref21] Piacenza E.; Bulgarini A.; Lampis S.; Vallini G.; Turner R. J. Biogenic SeNPs from *Bacillus mycoides* SeITE01 and *Stenotrophomonas maltophilia* SeITE02; Characterization with Reference to Their Associated Organic Coating. In AIP Conference Proceedings; AIP Publishing, 2017; p 020005.

[ref22] Piacenza E.; Presentato A.; Bardelli M.; Lampis S.; Vallini G.; Turner R. J. Influence of bacterial physiology on processing of selenite, biogenesis of nanomaterials and their thermodynamic stability. Molecules 2019, 24, 2532 10.3390/molecules24142532. 31373294 PMC6681009

[ref23] Cremonini E.; Boaretti M.; Vandecandelaere I.; Zonaro E.; Coenye T.; Lleo M. M.; Lampis S.; Vallini G. Biogenic selenium nanoparticles synthesized by *Stenotrophomonas maltophilia* SEITE02 loose antibacterial and antibiofilm efficacy as a result of the progressive alteration of their organic coating layer,”. Microb. Biotechnol. 2018, 11, 1037–1047. 10.1111/1751-7915.13260. 29635772 PMC6196382

[ref24] Piacenza E.; Presentato A.; Ferrante F.; Cavallaro G.; Alduina R.; Chillura Martino D. F. Biogenic selenium nanoparticles: a fine characterization to unveil their thermodynamic stability. Nanomaterials 2021, 11, 1195 10.3390/nano11051195. 34062748 PMC8147324

[ref25] Harrison J. J.; Rabiei M.; Turner R. J.; Badry E. A.; Sproule K. M.; Ceri H. Metal resistance in *Candida* biofilms. FEMS Microbiol. Ecol. 2006, 55, 479–491. 10.1111/j.1574-6941.2005.00045.x. 16466387

[ref26] Parasassi T.; Krasnowska E. K.; Bagatolli L.; Gratton E. Laurdan and Prodan as Polarity-Sensitive Fluorescent Membrane Probes. J. Fluoresc. 1998, 8, 365–373.

[ref27] Ames B. N. Assay of Inorganic Phosphate, Total Phosphate and Phosphatases. In Methods in Enzymology; Elsevier, 1966; Vol. 8, pp 115–118.

[ref28] Kerek E.; Hassanin M.; Prenner E. J. Inorganic mercury and cadmium induce rigidity in eukaryotic lipid extracts while mercury also ruptures red blood cells. Biochim. Biophys. Acta, Biomembr. 2018, 1860, 710–717. 10.1016/j.bbamem.2017.12.014. 29269315

[ref29] Daear W.; Mundle R.; Sule K.; Prenner E. J. The degree and position of phosphorylation determine the impact of toxic and trace metals on phosphoinositide containing model membranes. BBA Adv. 2021, 1, 10021 10.1016/j.bbadva.2021.100021. PMC10074965 37082006

[ref30] Sule K.; Prenner E. J. Lipid headgroup and side chain architecture determine manganese-induced dose dependent membrane rigidification and liposome size increase. Eur. Biophys. J. 2022, 51, 205–223. 10.1007/s00249-022-01589-x. 35166865

[ref31] Di Gregorio S.; Lampis S.; Vallini G. Selenite precipitation by a rhizospheric strain of *Stenotrophomonas* sp. isolated from the root system of *Astragalus bisulcatus*: a biotechnological perspective. Environ. Int. 2005, 31, 233–241. 10.1016/j.envint.2004.09.021. 15661289

[ref32] Srivastava N.; Mukhopadhyay M. Green synthesis and structural characterization of selenium nanoparticles and assessment of their antimicrobial property. Bioprocess Biosyst. Eng. 2015, 38, 1723–1730. 10.1007/s00449-015-1413-8. 25972036

[ref33] Xu C.; Guo Y.; Qiao L.; Ma L.; Cheng Y.; Roman A. Biogenic synthesis of novel functionalized selenium nanoparticles by *Lactobacillus casei* ATCC 393 and its protective effects on intestinal barrier dysfunction caused by enterotoxigenic *Escherichia coli* K88. Front. Microbiol. 2018, 9, 1129 10.3389/fmicb.2018.01129. 29967593 PMC6015882

[ref34] Piacenza E.; Campora S.; Carfì Pavia F.; Chillura Martino D. F.; Laudicina V. A.; Alduina R.; Turner R. J.; Zannoni D.; Presentato A. Tolerance, adaptation, and cell response elicited by *Micromonospora* sp. facing tellurite toxicity: a biological and physical-chemical characterization. Int. J. Mol. Sci. 2022, 23, 12631 10.3390/ijms232012631. 36293484 PMC9604092

[ref35] Suzuki M.; Lee D. Y.; Inyamah N.; Stadtman T. C.; Tjandra N. Solution NMR structure of selenium-binding protein from *Methanococcus vannielii*. J. Biol. Chem. 2008, 283, 25936–25943. 10.1074/jbc.M803773200. 18650445 PMC2533772

[ref36] Van Laer K.; Buts L.; Foloppe N.; Vertommen D.; Van Belle K.; Wahni K.; Roos G.; Nilsson L.; Mateos L. M.; Mamta R.; et al. Mycoredoxin-1 is one of the missing links in the oxidative stress defence mechanism of Mycobacteria. Mol. Microbiol. 2012, 86, 787–804. 10.1111/mmi.12030. 22970802

[ref37] Fujiwara K.; Toda H.; Ikeguchi M. Dependence of α-helical and β-sheet amino acid propensities on the overall protein fold type. BMC Struct. Biol. 2021, 12, 18 10.1186/1472-6807-12-18. PMC3495713 22857400

[ref38] Deponte M. Glutathione catalysis and the reaction mechanisms of glutathione-dependent enzymes. Biochim. Biophys. Acta, Gen. Subj. 2013, 1830, 3217–3266. 10.1016/j.bbagen.2012.09.018. 23036594

[ref39] Eberle R. J.; Kawai L. A.; de Morales F. R.; Tasic L.; Arni R. K.; Coronado M. A. Biochemical and biophysical characterization of a mycoredoxin protein glutaredoxin A1 from *Corynebacterium pseudotuberculosis*. Int. J. Biol. Macromol. 2018, 107, 1999–2007. 10.1016/j.ijbiomac.2017.10.063. 29042280

[ref40] Geng Z.; Song X.; Xing Z.; Geng J.; Zhang S.; Zhang X.; Wang Z. Effects of selenium on the structure and function of recombinant human S-adenosyl-L-methionine dependent arsenic (+3 oxidation state) methyltransferase in *E. coli*. JBIC, J. Biol. Inorg. Chem. 2009, 14, 485–496. 10.1007/s00775-008-0464-6. 19159958

[ref41] Kountouris P.; Hirst J. D. Predicting β-turns and their types using predicted backbone dihedral angles and secondary structures. BMC Bioinf. 2010, 11, 407 10.1186/1471-2105-11-407. PMC2920885 20673368

[ref42] Mendozza M.; Caselli L.; Salvatore A.; Montis C.; Berti D. Nanoparticles and organized lipid assemblies; from interaction to design of hybrid soft devices. Soft Matter 2019, 15, 8951–8970. 10.1039/c9sm01601e. 31680131

[ref43] Silvius J. R. Thermotropic Phase Transitions of Pure Lipids in Model Membranes and Their Modifications by Membrane Proteins. In Lipid-Protein Interactions; Silvius J. R., Ed.; John Wiley & Sons, Inc.: New York, USA, 1982.

[ref44] Chen X.; Tieleman D. P.; Liang Q. Modulating interactions between ligand-coated nanoparticles and phase-separated lipid bilayers by varying the ligand density and the surface charge. Nanoscale 2018, 10, 2481–2491. 10.1039/C7NR06494B. 29340405

[ref45] Chen X.; Tian F.; Zhang X.; Wang W. Internalization pathways of nanoparticles and their interaction with a vesicle. Soft Matter 2013, 9, 7592–7600. 10.1039/C3SM50931A.

[ref46] Dasgupta S.; Auth T.; Gompper G. Nano- and microparticles at fluid and biological interfaces. J. Phys.: Condens. Matter. 2017, 29, 373003 10.1088/1361-648X/aa7933. 28608781 PMC7104866

[ref47] Contini C.; Hindley J. W.; Macdonald T. J.; Barritt J. D.; Ces O.; Quirke N. Size dependency of gold nanoparticles interacting with model membranes. Commun. Chem. 2020, 3, 130 10.1038/s42004-020-00377-y. 33829115 PMC7610534

[ref48] Roiter Y.; Ornatska M.; Rammohan A. R.; Balakrishnan J.; Heine D. R.; Minko S. Interaction of nanoparticles with lipid membrane. Nano Lett. 2008, 8, 941–944. 10.1021/nl080080l. 18254602

[ref49] Le Bihan O.; Bonnafous P.; Marak L.; Bickel T.; Trepout S.; Mornet S.; De Haas F.; Talbot H.; Taveau J. C.; Lambert O. Cryo-electron tomography of nanoparticle transmigration into liposome. J. Struct. Biol. 2009, 168, 419–425. 10.1016/j.jsb.2009.07.006. 19596070

[ref50] Schneemilch M.; Quirke N. Free energy of adsorption of supported lipid bilayers from molecular dynamics simulation. Chem. Phys. Lett. 2016, 664, 199–204. 10.1016/j.cplett.2016.10.010.

[ref51] Arribas Perez M.; Moriones O. H.; Bastus N. G.; Puntes V.; Nelson A.; Beales P. A. Mechanomodulation of lipid membranes by weakly aggregating silver nanoparticles. Biochemistry 2019, 58, 4761–4773. 10.1021/acs.biochem.9b00390. 31508939

[ref52] Wang X.; Wang X.; Bai X.; Yan L.; Liu T.; Wang M.; Song Y.; Hu G.; Gu Z.; Miao Q.; Chen C. Nanoparticle ligand exchange and its effects at the nanoparticle-cell membrane interface. Nano Lett. 2019, 19, 8–18. 10.1021/acs.nanolett.8b02638. 30335394

[ref53] Van den Bogaart G.; Hermans N.; Krasnikov V.; de Vries A. H.; Poolman B. On the decrease in lateral mobility of phospholipids by sugars. Biophys. J. 2007, 92, 1598–1605. 10.1529/biophysj.106.096461. 17142271 PMC1796821

[ref54] Granjon T.; Vacheron M. J.; Vial C.; Buchet R. Mitochondrial creatine kinase binding to phospholipids decreases fluidity of membranes and promotes new lipid-induced β structures as monitored by Red Edge Excitation Shift, Laurdan fluorescence, and FTIR. Biochemistry 2001, 40, 6016–6026. 10.1021/bi002293e. 11352737

[ref55] Harrington J. M.; Chou H. T.; Gutsmann T.; Gelhaus C.; Stahlberg H.; Leippe M.; Armstrong P. B. Membrane activity of a C-reactive protein. FEBS Lett. 2009, 583, 1001–1005. 10.1016/j.febslet.2009.02.019. 19230837

[ref56] de Jonge M. I.; Pehau-Arnaudet G.; Fretz M. M.; Romain F.; Bottai D.; Brodin P.; Honore N.; Marchal G.; Jiskoot W.; England P.; Cole S. T.; Brosch R. ESAT-6 from *Mycobacterium tuberculosis* dissociates from its putative chaperone CFP-10 under acidic conditions and exhibits membrane-lysing activity. J. Bacteriol. 2007, 189, 6028–6034. 10.1128/JB.00469-07. 17557817 PMC1952024

[ref57] Zhang M.; Wang D.; Li P.; Sun C.; Xu R.; Geng Z.; Xu W.; Dai Z. Interaction of Hsp90 with phospholipid model membranes. Biochim. Biophys. Acta, Biomembr. 2018, 1860, 611–616. 10.1016/j.bbamem.2017.11.011. 29166573

[ref58] Leger A.; Azouz M.; Lecomte S.; Dole F.; Hocquellet A.; Chaignepain S.; Cabanne C. PiP2 favors an α-helical structure of non-recombinant Hsp12 of *Saccharomyces cerevisiae*. Protein Expression Purif. 2021, 181, 105830 10.1016/j.pep.2021.105830. 33485946

[ref59] Sum A. K.; Faller R.; de Pablo J. J. Molecular simulation study of phospholipid bilayers and insights of the interactions with disaccharides. Biophys. J. 2003, 85, 2830–2844. 10.1016/S0006-3495(03)74706-7. 14581188 PMC1303564

[ref60] Tan C.; Zhang Y.; Abbas S.; Feng B.; Zhang X.; Xia S.; Chang D. Insights into chitosan multiple functional properties: the role of chitosan conformation in the behavior of liposomal membrane. Food Funct. 2015, 6, 3702–3711. 10.1039/C5FO00256G. 26337678

[ref61] Cong L.; Wang J.; Lu H.; Tian M.; Ying R.; Huang M. Influence of different anionic polysaccharide coating on the properties and delivery performance of nanoliposomes for quercetin. Food Chem. 2023, 409, 135270 10.1016/j.foodchem.2022.135270. 36580701

[ref62] Wolfram J.; Suri K.; Yang Y.; Shen J.; Celia C.; Fresta M.; Zhao Y.; Shen H.; Ferrari M. Shrinkage of pegylated and non-pegylated liposomes in serum. Colloids Surf., B 2014, 114, 294–300. 10.1016/j.colsurfb.2013.10.009. PMC3884810 24216620

[ref63] Corbo C.; Molinaro R.; Taraballi F.; Furman N. T.; Sherman M. B.; Parodi A.; Salvatore F.; Tasciotti E. Effects of the protein corona on liposome-liposome and liposome-cell interactions. Int. J. Nanomed. 2016, 11, 3049–3063. 10.2147/IJN.S109059. PMC4938145 27445473

[ref64] Von White G. II; Chen Y.; Order-Hanna J.; Bothun G. D.; Kitchens C. L. Structural and thermal analysis of lipid vesicles encapsulating hydrophobic gold nanoparticles. ACS Nano 2012, 6, 4678–4685. 10.1021/nn2042016. 22632177

[ref65] Markowicz A.; Plociniczak T.; Piotrovska-Seget Z. Response of Bacteria to Heavy Metals Measured as Changes in FAME Profiles. Pol. J. Environ. Stud. 2010, 19, 957–965.

[ref66] Kaczorek E.; Salek K.; Guzik U.; Dudzinska-Bajorek B. Cell surface properties and fatty acids composition of *Stenotrophomonas maltophilia* under the influence of hydrophobic compounds and surfactants. New Biotechnol. 2013, 30, 173–182. 10.1016/j.nbt.2012.09.003. 22989923

