# Supplementary material for: Impact of Biogenic and Chemogenic Selenium Nanoparticles on Model Eukaryotic Lipid Membranes
Source: Langmuir. 2023 Jul 18;39(30):10406–19. doi: 10.1021/acs.langmuir.3c00718 (PMC10399287; doi:10.1021/acs.langmuir.3c00718)
Supplement: Supplementary file 1 — la3c00718_si_001.pdf [file la3c00718_si_001.pdf]

Supporting Information

Impact of biogenic and chemogenic selenium nanoparticles on model eukaryotic lipid membranes

Elena Piacenza[1,], Kevin Sule[2], Alessandro Presentato[1], Frieda Wells[2], Raymond Joseph Turner[2], Elmar J. Prenner[2,*]*

[1] Department of Biological, Chemical and Pharmaceutical Science and Technologies, University of Palermo, Viale delle Scienze, Ed. 16, 90128 Palermo, Italy; elena.piacenza@unipa.it (E.P.); alessandro.presentato@unipa.it (A.P.)

[2] Department of Biological Sciences, 2500 University Dr. NW, University of Calgary, Alberta, Canada, T2N 1N4; kevin.sule@ucalgary.ca (K.S.); frieda.wells@student.kuleuven.be (F.W.); turnerr@ucalgary.ca; (R.J.T.); eprenner@ucalgary.ca (E.J.P.)

*Corresponding Authors: elena.piacenza@unipa.it (E.P.); eprenner@ucalgary.ca (E.J.P.)

Table of Contents:

Table S1. ATR-FTIR absorption bands and identification of the bSeNP extract, cSeNPs, OM, and cSeNPs_OM.

Figure S1. Integral distribution of lipid $-CH_x$ stretching vibrations obtained for the analyzed samples from ATR-FTIR spectra.

Figure S2. Deconvolution of ATR-FTIR spectra of (**A-B**) bSeNP extract, (**C-D**) OM, and (**E-F**) cSeNPs_OM in the (**A,C,E**) 1780-1420 and (**B,D,F**) 1420-940 cm^{-1} regions.

Table S2. Deconvolution of ATR-FTIR spectra of the bSeNP extract, cSeNPs, OM, and cSeNPs_OM in the 1780-1420 cm^{-1} region.

Table S3. Deconvolution of ATR-FTIR spectra of the bSeNP extract, cSeNPs, OM, and cSeNPs_OM in the 1420-950 cm^{-1} region.

Figure S3. (**A**) Hydrodynamic diameter and (**B**) ζ potential values measured for yeast extract, POPC, and DMPC LUVs.

Table S1. ATR-FTIR absorption bands and identification of the bSeNP extract, cSeNPs, OM, and cSeNPs_OM.

ν **(cm^{-1})**				**Vibrational mode**	**Identification**
**bSeNP extract**	**OM**	**cSeNPs_OM**	**cSeNPs**		
3279	3280	3278		ν (NH)	Proteins (Amide A)[1]
3068	3067	3068		ν_s (NH^{3+})	Proteins (Amide B)[2]
			3049	ν (CH_2)	RSOSR residues and RSO_2^-, RSSR adsorbed on SeNP[3]
			2980	ν (CH)	RSSR residues[3]
2957	2956	2956		ν_{as} (CH_3)	Fatty acids[1,2,4]
2925	2924	2925	2940	ν_{as} CH_2; ν (CH)	Fatty acids; RSO_2^-, RSSR residues and RSSR adsorbed on SeNP[1-4]
			2900	ν (CH); ν_s (CH_2)	RSSR residues[3]
2872	2871	2871	2877	ν_s (CH)	Amino acids in fatty acids; RSSR residues[3,4]
2855	2854	2852	2840	ν_s (CH_2)	Fatty acids; RSSR residues[1-4]
			2711	ν (NH_2)	RSSR residues[3]
1743	1742	1742		ν (CO)	Ester moieties of lipids and polysaccharides[1,2,4]
1651	1648	1652		ν (CO); δ (NH)	Random coil proteins (Amide I)[5]
1630	1631	1629		ν (CO); δ (NH)	β-sheet proteins (Amide I)[1]
			1612(s)	δ (NH_2)	RSSR, RSOSR, residues and RSSR adsorbed on SeNP[3]
			1586	δ_{as} (NH_3^+)	RSSR residues[3]
1533	1534	1534		δ (NH); ν (CN)	Amide II of proteins (Amide II)[1,2,4]
1518	1517	1518	1511	δ_s (NH_3^+)	Amino acid residues; RSSR residues[2,3]
1467(s)	1469(s)	1468(s)	1461	δ (CH_2); δ (CH_3); β (CH_2); δ (OH)	Lipids and proteins; RSSR adsorbed on SeNP[3,4]
1452	1454	1455		δ_{sciss} (CH_2); δ (OH); ν CC(O)	Polysaccharides; aliphatic groups of proteins[6,7]
1437(s)	1436(s)	1436(s)		δ_{sciss} (CH_2); δ (OH); ν CC(O); ν_s (COO^-)	Polysaccharides; aliphatic groups of proteins[6,7]
			1424	δ (CH); δ (CH_2)	RSSR residues and RSO_2^-, RSSR, adsorbed on SeNP[3]
1397(s)	1395(s)	1395(s)	1395	ν_s (COO^-); δ (OH); δ (CH); δ (NH_2)	Amino acid side chains; free fatty acids; RSO_2^-, RSSR, RSOSR residues and RSO_2^- adsorbed on SeNP[3,4]
1386	1385	1385		δ (CH); δ (OH); δ (COH); β (CH_3); ν CC(O); ν (CN) ν_s (COO^-); δ (NH_2)	Aldehydes; carboxylic acids; peptides; aromatic amines[7,8]
1367	1368	1367		β (CH_3); δ (CH); ν_s (COO^-)	Lipids and proteins[4]
1340(s)	1339(s)	1341(s)	1345(s)	β C(OH); ν_s (COO^-); δ (OH); δ (CH)	Polysaccharides; RSO_2^-, RSSR, RSOSR residues[3-5]
1310	1308	1309	1307	ν (C-OH); δ (OH); δ (CH); ω (CH_2)	Polysaccharides in EPS; RSOSR residues and RSO_2^-, RSSR adsorbed on SeNP[3,9]
			1294	δ (OH); ω (CH_2); δ (CH_2); δ (CH)	RSO_2^-, RSSR residues[3]

			1262(s)	δ (CH_2); δ (OH); δ (NH_2)	RSSR, RSOSR, residues and RSO_2^-, RSSR adsorbed on SeNP[3]
1232	1231	1231		ν (CN); δ (NH_2); β (NH); ν_{as} (PO_2^-)	Proteins (Amide III); Nucleic acids[1,4]
			1201	δ (OH); δ (CH_2)	RSO_2^-, RSSR residues[3]
			1179	δ (S)OH; δ (CH_2); δ (OH)	RSSR, RSOSR, residues and RSO_2^-, RSSR adsorbed on SeNP[3]
1160	1158	1161		ν_{as} (COC); δ (CH_2); δ (CH); δ (NH_2); ν (CO); δ (OH)	Nucleic acids; $\alpha_{(1,4)}$ glycosidic bonds; polysaccharide ring; amino acids[10-12]
			1138	δ (CH); δ (CH_2); δ (S)OH	RSSR, RSOSR, residues and RSO_2^- adsorbed on SeNP[3]
1122(s)	1122(s)	1123(s)		δ_{ip} (OH); ν_{as} (COC); δ (CH_2); δ (CH)	Nucleic acids; $\alpha_{(1,4)}$ glycosidic bonds; polysaccharide ring[10,11]
			1102	ω (CH_2); δ (CH_2); δ (S)OH	RSO_2^-, RSSR, residues and RSSR adsorbed on SeNP[3]
1063	1064	1062	1062	ν (CO); ν (CC); ν (COH); δ (COC); ρ (NH_3^+); ν (SO); δ (CH_2)	Polysaccharides, proteins, and polyesters; amino acid residues; RSSR residues and RSO_2^- adsorbed on SeNP[3,13,14]
1027(s)	1030(s)	1026(s)		ν (PO)	Polysaccharides and nucleic acids[1,2,4,5]
			1015	δ (NH_2)	RSOSR residues[3]
980	981	979	987	δ (NH_2); ν (CC); ν (CO); δ (HNCC); ν_s (PO_3^{2-}); δ (COH)	Amino acids; $\beta_{(1,3)}$ glycosidic bonds; nucleic acids; polysaccharides; RSO_2^- adsorbed on SeNP[3,10-15]
			961	δ (NH_2); δ (CH_2)	RSO_2^- adsorbed on SeNP[3]
927	925	926	935	δ (=CH); τ (CH_2); δ (CH_2); ν_s (PO_4^{3-}); δ (NH_2)	Alkyl halides, carboxylic acids; amines; $\alpha_{(1,3)}$ glycosidic bonds; amino acid residues; nucleic acids; RSSR residues[3,6,10,12]
			915	δ (NH_2); ν (SO)	RSO_2^-, RSSR residues[3]
			902	δ (NH_2); δ (CH_2)	RSOSR residues[3]
			876	δ (NH_2); δ (CH_2)	RSO_2^- adsorbed on SeNP[3]
			870	δ (NH_2); δ (CH_2)	RSSR, RSOSR residues[3]
837	838	835	841	ν (CC); ν (CN); δ (NH_2)	Amino acid residues; RSOSR residues[3,12]
			821	δ (NH_2); ν_s (SeO); δ (CH_2)	Na_2SeO_3, RSO_2^-, RSSR, residues and RSO_2^- adsorbed on SeNP[3,16]
			800	ν SO; δ (OH); δ (NH_2)	RSSR, RSOSR residues and RSSR adsorbed on SeNP[3]

$\tilde{\nu}$, ν, δ, β, ω, ρ, and τ indicate wavenumber, stretching, bending, deformation, wagging, rocking, and twisting, respectively; s, as, sciss, and ip stand for symmetric, asymmetric, scissoring, and in-plane vibrations.

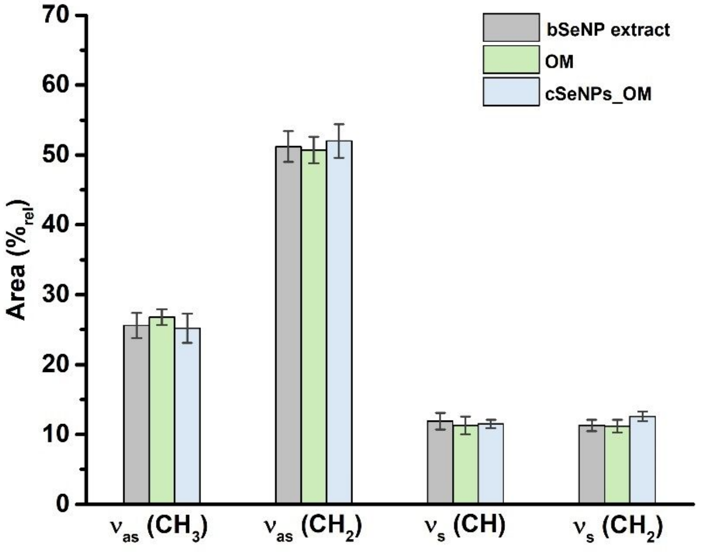

Figure S1. Integral distribution of lipid -CH_x stretching vibrations obtained for the analyzed samples from ATR-FTIR spectra.

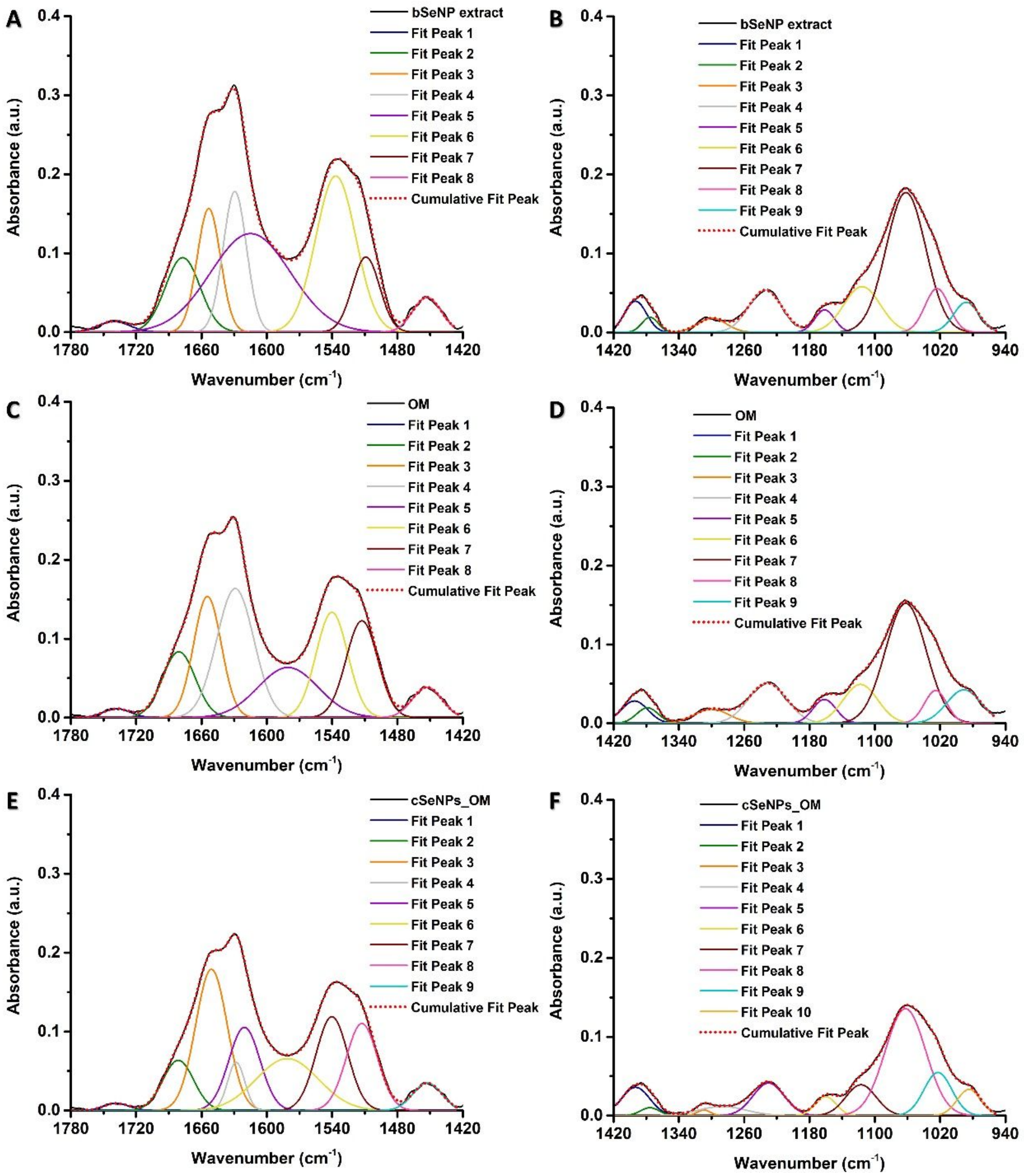

Figure S2. Deconvolution of ATR-FTIR spectra of (**A-B**) bSeNP extract, (**C-D**) OM, and (**E-F**) cSeNPs_OM in the (**A,C,E**) 1780-1420 and (**B,D,F**) 1420-940 cm^{-1} regions.

Table S2. Deconvolution of ATR-FTIR spectra of the bSeNP extract, cSeNPs, OM, and cSeNPs_OM in the 1780-1420 cm^{-1} region.

bSeNP extract [$\tilde{\nu}$ (cm^{-1})][a]	w	A	OM [$\tilde{\nu}$ (cm^{-1})][b]	w	A	cSeNPs_OM [$\tilde{\nu}$ (cm^{-1})][c]	w	A	Vibrational mode
1740	29.1±3.1	0.49±0.04	1738	26.7±1.3	0.35±0.01	1738	23.5±1.4	0.27±0.01	ν (CO)[1,2,4]
			1681	29.9±1.9	3.14±0.21	1681	28.3±2.5	2.26±0.59	ν (CO); δ (NH)[3]
1672	31.9±3.2	3.78±0.23							ν (CO); δ (NH)[3]
1653	21.5±1.2	4.21±0.47	1654	25.5±1.9	4.91±0.18	1650	29.1±1.9	6.52±0.62	ν (CO); δ (NH)[5]
1629	21.4±1.1	4.79±0.46	1629	34.2±0.9	7.00±0.53	1628	15.7±0.2	1.20±0.04	ν (CO); δ (NH)[1]
1615	73.3±3.6	11.5±2.1				1620	28.6±1.5	3.78±0.34	ν (CO); δ (NH)[1]
			1572	55.2±3.9	4.40±0.41	1575	56.9±3.2	4.70±0.66	ν_{as} (COO^-)[3]
1537	36.8±1.7	9.13±0.81	1540	29.1±1.6	4.87±0.65	1540	29.2±1.3	4.34±0.12	δ (NH); ν (CN)[1,2,4]
1509	25.1±0.8	2.99±0.21	1512	29.1±0.7	4.45±0.32	1512	28.9±0.4	3.99±0.15	δ_s (NH_3^+)[2]
1453	25.9±0.5	1.44±0.02	1453	27.1±0.4	1.32±0.02	1453	26.5±0.4	1.16±0.02	δ_{sciss} (CH_2); δ (OH); ν CC(O)[6,7]

$\tilde{\nu}$, ν, δ, s, as, and sciss indicate wavenumber, stretching, bending, symmetric, asymmetric, and scissoring vibrations, respectively.

[a] r-χ^2 = $6.28\cdot10^{-6}$ R^2 = 0.9996; [b] r-χ^2 = $2.60\cdot10^{-6}$ R^2 = 0.9995; [c] r-χ^2 = $2.32\cdot10^{-6}$ R^2 = 0.9995

Table S3. Deconvolution of ATR-FTIR spectra of the bSeNP extract, cSeNPs, OM, and cSeNPs_OM in the 1420-950 cm^{-1} region.

bSeNP extract [$\tilde{\nu}$ (cm^{-1})][a]	w	A	OM [$\tilde{\nu}$ (cm^{-1})][b]	w	A	cSeNPs_OM [$\tilde{\nu}$ (cm^{-1})][c]	w	A	Vibrational mode
1393	26.8±2.1	1.33±0.22	1394	32.6±2.4	1.16±0.02	1392	34.4±1.9	1.55±0.32	ν_s (COO^-); δ (OH); δ (CH); δ (NH_2)[3,4]
1375	19.9±0.5	0.48±0.02	1378	26.6±1.2	0.66±0.05	1376	23.3±0.9	0.29±0.02	β (CH_3); δ (CH); ν_s (COO^-)[4,7,8]
1299	35.2±0.9	0.78±0.01	1299	42.5±0.9	0.96±0.02	1305	16.5±1.2	0.15±0.02	ν (C-OH); δ (OH); δ (CH); ω (CH_2)[3,9]
						1287	58.3±3.7	0.85±0.05	δ (CH_2); δ (OH); δ (NH_2)[3]
1234	40.8±0.3	2.74±0.03	1231	46.6±0.4	2.97±0.03	1230	38.3±0.4	1.99±0.05	ν (CN); δ (NH_2); β (NH); ν_{as} (PO_2^-)[1,4]
			1162	28.5±0.8	1.07±0.07	1160	25.6±0.5	0.77±0.03	ν_{as} (COC); δ (CH_2); δ (CH); δ (OH); δ (NH_2); ν (CO)[10-12]
1116	24.3±0.8	0.87±0.05	1117	42.2±2.7	2.60±0.32	1117	37.1±1.9	1.84±0.05	δ_{ip} (OH); ν_{as} (COC); δ (CH_2); δ (CH)[10,11]
1062	48.5±2.6	10.8±1.1	1062	50.2±3.1	9.56±0.62	1063	49.6±2.1	8.43±0.62	ν (CO); ν (CC); ν (COH); δ (COC); ρ (NH_3^+); ν (SO); δ (CH_2)[3,13,14]
1023	29.6±2.2	2.06±0.08	1025	27.6±1.6	1.43±0.08	1023	34.0±1.8	2.33±0.12	ν (PO)[1,2,4,5]
988	30.7±1.2	1.47±0.06	990	42.3±1.3	2.25±0.06	986	29.1±0.3	1.22±0.05	δ (NH_2); ν (CC); ν (CO); δ (COH); δ (HNCC); ν_s (PO_3^{2-})[3,10-15]

$\tilde{\nu}$, ν, δ, β, ω, ρ, s, as, sciss, and ip indicate wavenumber, stretching, bending, wagging, deformation, rocking, symmetric, asymmetric, scissoring, and in-plane vibrations, respectively.

[a] r-χ^2 = $2.04 \cdot 10^{-6}$ R^2 = 0.9992; [b] r-χ^2 = $1.12 \cdot 10^{-6}$ R^2 = 0.9993; [c] r-χ^2 = $7.05 \cdot 10^{-7}$ R^2 = 0.9995

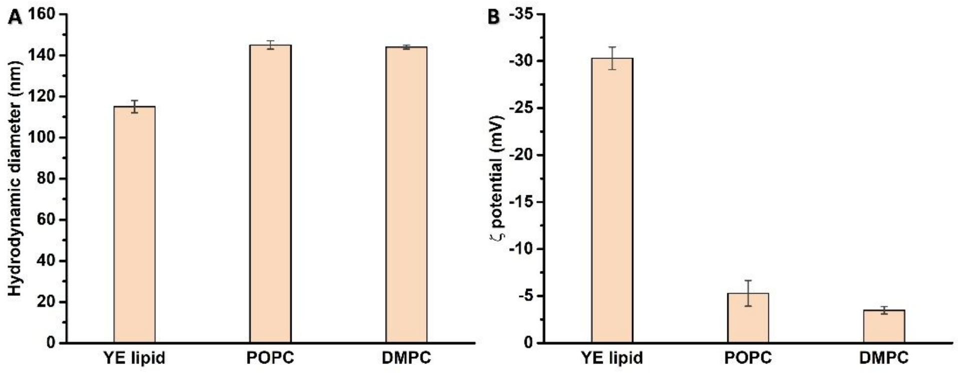

Figure S3. (**A**) Hydrodynamic diameter and (**B**) ζ potential values measured for yeast extract, POPC, and DMPC LUVs.

REFERENCES

1. Tugarova, A.V.; Mamchenkova, P.V.; Dylatova, Y.A.; Kamnev, A.A. FTIR and Raman spectroscopic studies of selenium nanoparticles synthesized by the bacterium *Azospirillum thiophilum*. *Spectrochim. Acta A. Mol. Biomol. Spectrosc*. **2018**, *192*, 458–463, doi: 10.1016/j.saa.2017.11.050

2. Faghihzadeh, F.; Anaya, N.M.; Schifman, L.A.; Oyanedel-Craver, V. Fourier transform infrared spectroscopy to assess molecular-level changes in microorganisms exposed to nanoparticles. *Nanotechnol. Environ. Eng.* **2016**, *1*, 1, doi: 10.1007/s41204-016-0001-8

3. Piacenza, E.; Presentato, A.; Ferrante, F.; Cavallaro, G.; Alduina, R.; Chillura Martino, D.F. Biogenic selenium nanoparticles: a fine characterization to unveil their thermodynamic stability. *Nanomaterials*, **2021**, *11*, 1195, doi: 10.3390/nano11051195

4. Lasch, P.; Naumann, D. Infrared spectroscopy in microbiology. In: *Encyclopedia of Analytical Chemistry*. Meyers, R.A., Ed.; Wiley Online Library, 2015, 10.1002/9780470027318.a0117.pub2

5. Wang, X.; Wang, W.; Liu, P.; Wang, P.; Zhang, L. Photocatalytic Degradation of E.Coli Membrane Cell in the Presence of ZnO Nanowires. *J. Wuhan Univ. Technol. Mater. Sci. Ed.* **2011**, *26*, 222-225, doi:10.1007/S11595-011-0201-9.

6. Jiang, W.; Saxena, A.; Song, B.; Ward, B.B.; Beveridge, T.J.; Myneni, S.C.B. Elucidation of functional groups on Gram-positive and Gram-negative bacterial surfaces using Infrared spectroscopy. *Langmuir* **2004**, *20*, 11433-11442, doi: 10.1021/la049043+

7. Buszewski, B.; Dziubakiewicz, E.; Pomastowski, P.; Hrynkiewicz, K.; Ploszaj-Pyrek, J.; Talik, E.; Kramer, M.; Albert, K. Assignment of functional groups in Gram-positive bacteria. *J. Anal. Bioanal. Tech.* **2015**, *6*, 1, doi: 10.4172/2155-9872.1000232

8. Otari, S.V.; Patil, R.M.; Ghosh, S.J.; Thorat, N.D.; Pawar, S.H. Intracellular synthesis of silver nanoparticle by actinobacteria and its antimicrobial activity. *Spectrochim. Acta A Mol. Biomol. Spectrosc.* **2015**, *136,* 1175-1180, doi: 10.1016/j.saa.2014.10.003

9. Kepenek, E.S.; Gozen, A.G.; Severcan, F. Molecular Characterization of Acutely and Gradually Heavy Metal Acclimated Aquatic Bacteria by FTIR Spectraoscopy. *J. Biophotonics* **2019**, *12*, doi: 10.1002/jbio.201800301

10. Nikonenko, N.A.; Buslov, D.K., Sushko, N.J.; Zhbankov, R.G. Investigation of stretching vibrations of glycosidic linkages in disaccharides and polysaccharides with use of IR spectra deconvolution. *Biopolymers* **2000**, *57*, 257-262, 10.1002/1097-0282(2000)57:4<257::AID-BIP7>3.0.CO;2-3

11. Nadtochenko, V.A.; Rincon, A.G.; Stanca, S.E., Kiwi, J. Dynamics of *E. coli* membrane cell peroxidation during TiO_2 photocatalysis studied by ATR-FTIR spectroscopy and AFM microscopy. *J. Photochem. Photobiol. A Chem.* **2005**, *169*, 131-137, doi: 10.1016/j.jphotochem.2004.06.011

12. Mohamed, M.E.; Mohammed, A.M.A. Experimental and computation vibration study of amino acids. *Inter. Lett. Chem. Phys. Astr.* **2013**, *10*, 1-17, doi: 10.18052/www.scipress.com/ILCPA.15.1

13. Kamnev, A.A.; Mamchenkova, P. v.; Dyatlova, Y.A.; Tugarova, A. v.; Kamnev, A.A.; Mamchenkova, P. v.; Dyatlova, Y.A.; Tugarova, A. v. FTIR Spectroscopic Studies of Selenite Reduction by Cells of the Rhizobacterium Azospirillum Brasilense Sp7 and the Formation of Selenium Nanoparticles. *J. Mol. Struct.* **2017**, *1140*, 106-112, doi: 10.1016/j.molstruc.2016.12.003
14. Kurihara, T.; Noda, Y.; Takegoshi, K. Capping Structure of Ligand-Cysteine on CdSe Magic-Sized Clusters. *ACS Omega* **2019**, *4*, 3476-3483, doi: 10.1021/acsomega.8b02752
15. Barth, A. The infrared absorption of amino acid side chains. *Progr. Biophys. Mol. Biol.* **2000**, *74*, 141-173, doi; 10.1016/S0079-6107(00)00021-3
16. Makatun, V.N.; Pechkovskii, V.V.; Melnikova, R.Ya.; Gusev, S.S. Infrared spectra of copper selenites. *Zhurnal Prikladnoi Spektroscopii*. **1970**, *12*, 497-503.
